# Impact of sperm fractionation on chromosome positioning, chromatin integrity, DNA methylation, and hydroxymethylation level

**DOI:** 10.1186/s11658-025-00830-7

**Published:** 2025-12-23

**Authors:** Zuzanna Graczyk, Jagoda Kostyk, Julia Pospieszna, Zuzanna Myslicka, Marzena Kamieniczna, Monika Fraczek, Marta Olszewska, Maciej Kurpisz

**Affiliations:** 1 Institute of Human Genetics, Polish Academy of Sciences, Strzeszynska 32, 60-479 Poznan, Poland; 2 Department of Toxicology, Poznan University of Medical Sciences, Collegium Pharmaceuticum, Rokietnicka 3, 60-806 Poznan, Poland

**Keywords:** Sperm chromosomes, Nuclear order, Sperm methylation, Sperm hydroxymethylation, Sperm fractions, Sperm DNA fragmentation, Sperm chromatin, Male infertility, Swim up, Density gradient

## Abstract

**Background:**

Sperm chromosomes are nonrandomly organized in the cell nucleus, which plays an important role in the regulation of early embryo development, which is determined by the specific localization of sperm chromosomal regions carrying genes with expression crucial at the first contact with ooplasm during fertilization. Thus, the aim of this study is to determine whether the application of selective methods providing high-quality spermatozoa with good motility and/or morphology can increase the frequency of gametes with a specific positioning of chromosomes. For the first time, we used a sequential staining algorithm for consecutive analyses of the same individual spermatozoon with a fixed position, what enables one to achieve full and detailed documentation at the single cell level.

**Methods:**

Semen samples from five normozoospermic males were collected and processed for fractionation via swim up (to select viable and motile spermatozoa) or Percoll density gradient (90%/47%; to select viable sperm with normal motility and morphology). Sperm chromatin protamination was assessed by Aniline Blue (AB) staining, and DNA fragmentation by Acridine Orange (AO) (ssDNA fragmentation) or terminal deoxynucleotidyl transferase dUTP nick end labeling (TUNEL) assay (ssDNA and dsDNA fragmentation). Then, sequential staining and analyses of the same individual spermatozoon with a fixed position on a slide were performed, in the following order: (i) fluorescence in situ hybridization (FISH) for determination of positioning of chromosomal centromeres: 4, 7, 8, 9, 18, X, and Y, with so-called linear and radial estimations applied, followed by distance measurements between selected pairs of chromosomes; and (ii) immunofluorescent (IF) measurement of global sperm DNA methylation (5mC) and hydroxymethylation (5hmC) levels, which added additional data about the epigenetic layer of the sperm chromosomes’ positioning.

**Results:**

Our study demonstrated that high-quality sperm selection methods significantly: (i) increased the frequency of spermatozoa with good chromatin protamination (+ ~25%) and 5mC and 5hmC DNA levels (+ ~9.5%) and (ii) reduced the rate of spermatozoa with ssDNA fragmentation (− ~65%). Motile and morphologically normal spermatozoa showed distinct chromosome repositioning with sex chromosomes shifted to the nuclear periphery, a key chromosomal region of the initial interaction with the ooplasm during fertilization process. Evaluated autosomes revealed various patterns of repositioning.

**Conclusions:**

Our findings underline the validity of methods used for selection of high-quality spermatozoa in assisted reproductive technologies (ART), also in the context of the sperm chromosomal topology and chromatin integrity, crucial at the first steps during fertilization.

**Supplementary Information:**

The online version contains supplementary material available at 10.1186/s11658-025-00830-7.

## Introduction

Infertility is a significant global health issue, affecting approximately 10–18% of couples of reproductive age, with male background contributing to 40–60% of all cases [1]. Male infertility can result from various environmental factors, including tobacco smoking, drug use, anabolic steroids, alcohol consumption, followed by exposure to heavy metals, radiation, organic chemicals, or high temperatures [2]. Genomic and cytogenomic aberrations account for approximately 10–15% of male infertility cases [3–5]. Importantly, approximately 50% of genetic causes are strictly linked to spermatogenesis disruptions, underlining the high genetic vulnerability of the process of male gamete generation—spermatozoa with a haploid genome. Their task is to deliver to the oocyte the paternal genetic material important for the development of the embryo [6, 7].

The chromatin organization of spermatozoa differs from chromatin packaging in somatic cells. Mainly, during spermatogenesis, the sperm chromatin becomes drastically compacted and also transcriptionally silenced at an extremely high level [8–10]. This chromatin rearrangement occurs because of an exchange of at least 85% of the somatic type of histones: first, by transition proteins (TPs), and then by the protamines, leading to the formation of DNA–protamine complexes (toroids) with strongly condensed chromatin—about sixfold more compact than in somatic cells [8, 11, 12]. This unique chromatin organization observed in the sperm nucleus likely serves several critical functions, including: (i) efficient compaction of paternal DNA, to protect the genome from potential damage while passaging through the female reproductive tract [13]; (ii) effective mass silencing of sperm gene expression, but with preserved capacity for rapid reactivation of genes following fertilization [14]; and (iii) efficient repair of DNA damage and remodeling of the paternal chromatin, critical for early embryonic divisions through the sequential order of the paternal genome delivering to the maternal oocyte [8, 15]. It has also been clearly shown that abnormalities in histone transition and protamine amount in humans may impair spermatogenesis and compromise sperm chromatin remodeling. The latter process is finalized during epididymal maturation, when disulfide bonds are formed to stabilize the final chromatin structure. Such defects may contribute to reduced sperm quality, male infertility, and unsuccessful attempts at in vitro fertilization using assisted reproductive technologies (ART) [9, 10, 16–18].

It has been documented that the location of the chromosomes in cell nucleus is well-defined and nonrandom within the so-called chromosome territories (CTs). CTs together with topologically associating domains (TADs) and interchromatin compartments (ICs) interact with the elements of the nuclear matrix, creating the intranuclear architecture [19–22]. Therefore, the topology of chromosomes may have specific characteristics by determining the location of their centromeres and/or the CTs, as well as the p and q arms of chromosomes [22]. For somatic cells, there is a variety of novel techniques with ultrahigh resolution (i.e., GAM, Hi-C, SPRITE, ChIA-Drop) for exploring TADs and all possible linkages between particular regions of the chromosomes [15, 22]. In the case of spermatozoa, the visualization of chromosomes still relies on fluorescent in situ hybridization (FISH) with probes specific for particular parts of the chromosome (arms, centromeres, bands, (sub)telomere regions, etc.) as spermatozoa remain virtually transcriptionally inactive, limiting the applicability of techniques that depend on RNA or active transcriptional processes [12, 15, 23, 24]. Determination of the organization of chromosomes in the human sperm nucleus is based mostly on the linear and radial localization of centromeres [8, 11, 12, 23, 25, 26] that allow an estimate of their positioning in a preferential part of the nucleus, in relation to the sperm apical, central, and basal regions, as well as the localization’s depth in a three-dimensional (3D) manner.

It has also been suggested that the sperm nucleus architecture plays an important role in the regulation of early embryo development via the first contact with ooplasm of the chromosomal regions carrying specific genes whose expression is crucial at the first stages of embryo development [6, 15, 25]. For instance, in humans, the sperm X chromosome is typically positioned within the apical region of the sperm nucleus [12, 27–29]. This localization may be important for rapid activation of genes immediately after the fusion of gametes. A subset of genes located on sex chromosomes (e.g., *RPS4Y1* (gene ID 6192) and *RPS4X* (gene ID 6191)) encode ribosomal proteins that are expressed soon after fertilization and may indirectly influence the regulation of autosomal genes during early embryonic development [30–34]. Given their regulatory roles, it is likely that sex chromosomes are transcribed first, as they influence the expression of autosomal genes (e.g. *KDM6A* (gene ID 7403), *SRY* (gene ID 6736)) essential for initiating proper regulatory networks and ensuring correct embryonic development [30, 31, 34]. The development of sex-specific regulatory networks enriched in X- and Y-linked genes plays a critical role in cellular differentiation, also during early mammalian development [35–37]. Moreover, female embryos undergo X chromosome inactivation (XCI) during implantation, to balance the X-linked gene dosage between XY and XX embryos [38–44]. Impaired XCI is one of the major epigenetic barriers that can hinder correct development of female embryos, often resulting in early miscarriage and embryonic lethality [45–47]. In humans, after the completion of embryonic genome activation at E4, female cells possess two active X chromosomes [48]. Both X chromosomes in females are widely activated immediately after embryo genome activation from the four‐ to eight‐cell stage. Importantly, during early embryogenesis, the sex chromosomes display distinct activation patterns: only a limited number of Y-linked genes are initially expressed, whereas a broader region of the X chromosome shows transcriptional activity [34]. For example, *RPS4Y1* (gene ID 6192), a gene located on the Y chromosome, is highly expressed during embryonic genome activation and shows a sex-specific pattern. Its paralog, *RPS4X* (gene ID 6191), is present on a long arm of the X chromosome (Xq) [49]. *RPS4X* is also known to escape from X inactivation [49]. It has been assumed that normal human development requires two *RPS4* genes per cell: two copies of *RPS4X* in female cells, and one copy of *RPS4X* and one copy of *RPS4Y* in male cells [34]. Transcription of *RPS4Y1* helps balance the gene dosage between sexes during early development, compensating for the twofold dosage of *RPS4X* in females after embryonic genome activation, as both X chromosomes remain active [50].

Another example supporting the importance of spermatozoon nucleus architecture, as well as the observation that X chromosomes are typically positioned within the apical region of the sperm nucleus, which may facilitate the rapid activation of genes immediately after gamete fusion, is the *SMC1A* gene (gene ID 8243) located on the X chromosome, in an area that escapes XCI [51]. *SMC1A* encodes a structural component of the cohesin complex, which ensures proper cohesion of sister chromatids in mitosis and meiosis [52]. This is crucial for the correct segregation of chromosomes during cell division. The encoded protein is also thought to be an important part of functional kinetochores [51]. In addition, this protein has a potential role in DNA repair and genome stability maintenance [53–56]. Furthermore, together with *CTCF* transcription factor (gene ID 10,664), *SMC1A* takes part in organizing the three-dimensional structure of the genome also in pre-implantation embryo development [57–60].

These examples seem to highlight the significance of X- or Y-located genes delivered by spermatozoa, underlining the importance of the sex chromosomes’ spatial organization within the sperm nucleus, especially considering that the activation of sex chromosome-linked regulatory networks is known to be critical for early embryonic development and cellular differentiation [34, 61]. Adapting of ultrahigh-resolution techniques such as GAM or Hi-C for use in the evaluation of spermatozoa could significantly improve our understanding of the unique organization of the sperm nucleus. However, as mentioned above, the almost transcriptionally inactive nature of spermatozoa limits the applicability of methods relying on RNA or active transcriptional processes, which explains the current reliance on FISH for chromosomal visualization [62, 63]. However, a promising direction for future studies in this field is that the implementation of such cutting-edge techniques in sperm research would help reveal the specific interactions between chromosomal regions and shed light on how the nuclear architecture contributes to the regulation of early embryonic development.

Another issue that is crucial both for sperm characteristics as well as for fertilization or early embryo development events is epigenetic modifications of sperm DNA or histones [58, 64–68]. It has been found that sperm DNA methylation (5mC) and DNA hydroxymethylation (5hmC) can affect the results of ART and are linked to embryonic development [69, 70]. Spermatozoa, which in principle are transcriptionally inactive, exhibit distinct methylation patterns characterized by the global hypermethylation of sperm DNA in contrast to hypomethylated somatic cells. This hypermethylation is predominantly observed in intergenic regions and repetitive elements, contributing to support genomic stability [66]. The appropriate parent-of-origin expression in the developing embryo strictly relies on the hypermethylation of imprinted genes [13, 67, 71, 72]. On the other hand, promoters of developmental genes and imprinting control regions are specifically hypomethylated, which is essential for proper gene expression during early embryonic development. This hypomethylation facilitates the instant activation of developmental genes, leading to epigenetic reprogramming and developmental assignment determination [13, 58, 65, 67, 68, 73–75]. Understanding these sperm-specific epigenetic patterns and their regulatory consequences is crucial for elucidating the mechanisms of transgenerational epigenetic inheritance and their impact on offspring health.

Recent studies have shown that environmentally induced parental epigenetic alterations can be transmitted to future generations and influence the phenotype of the offspring and their potential disease development [76–80]. When focusing on DNA methylation, it is well-established that it is crucial for processes such as genetic imprinting, gene silencing, chromosome X inactivation, and protein conformational changes [67, 71, 73, 81–83]. Parental genomes have distinct genetic roles after fertilization, driven by gametic imprinting and unique methylation patterns established during gametogenesis [13, 67, 71, 72, 84]. The paternal genome primarily supports early placental development, while the maternal genome governs embryonic development [13, 67, 72, 84]. Developmental disturbances in embryos may arise from improper activation of key genes, often linked to disrupted methylation/demethylation cycles in gametogenic cells [13, 67, 68, 74, 85–87]. Hydroxymethylation (5hmC) occurs at lower levels (0.1–0.8%) compared with methylation, with higher values in tissues with active transcription, such as neurons [83, 88–90]. This epimark, derived from enzymatic oxidation of 5mC, is found in enhancers, gene promoters, and regulatory elements [83, 91]. In transcriptionally inactive spermatozoa, 5hmC levels are about fourfold lower than in somatic cells [92]. Together with Tet enzymes (ten–eleven translocation proteins; Tet1, Tet2, and Tet3), 5hmC may regulate gene expression by modulating methylation, highlighting the critical roles of both 5mC and 5hmC in genome function [88, 90, 92–95].

Sperm fractionation is routinely utilized in infertility ART treatment clinics, e.g., for in vitro fertilization (IVF) [96] or intracytoplasmic sperm injection (ICSI) [97]. Some of the well-known methods of sperm separations are swim-up fractionation or density-gradient centrifugation [69]. In the swim-up (SU) method, the semen is centrifuged to separate cells from the seminal fluid, and the pellet, containing all types of spermatozoa, cells, and debris, is placed into a tube containing a culture medium, then the motile spermatozoa that swim to the upper border are used in ART treatments. In the density-gradient centrifugation (DGC) method, the semen is centrifuged along density columns to allow the separation of morphologically normal and motile human spermatozoa, free of debris, dead cells, and nongerm cells [98]. Spermatozoa obtained by these two methods result in similar cumulative live birth rates [99]. In advanced ART centers, the semen is subjected to DGC and then SU [100].

Evidence has shown that selection of spermatozoon before injection into the oocyte has a significant impact on ICSI outcomes [101]. Choosing proper spermatozoa remains a pivotal stage, influencing fertilization processes and early embryo development. Nevertheless, the main challenge arises from the varying quality of spermatozoa within an ejaculate [102]. The zona pellucida of the oocyte acts as a selective barrier during fertilization and prevents penetration of multiple spermatozoa. During ICSI or intracytoplasmic morphologically selected sperm injection (IMSI), the best spermatozoa for oocyte microinjection are selected according to their correct shape and motility [103]. However, such visual inspection for normal spermatozoa does not eliminate the genetically abnormal spermatozoa—as visually normal spermatozoa can be (and often are) carriers of genetic or epigenetic mutations that may disturb fertilization events or embryo development or be devastating for the offspring. Therefore, it is important to deepen knowledge on the mechanisms influencing sperm quality to improve the selection process for broadly used in vitro fertilization.

In this context, the aim of this study is to investigate for the first time whether chromosomal localization differs in good-quality fractions of human spermatozoa separated via two different protocols: SU or DGC, followed by screening of sperm DNA methylation and hydroxymethylation levels. This aim was achieved by sequential staining algorithm of the same individual sperm with a fixed position on a slide, in a given order: (i) determination of chromosomal positioning of centromeres 4, 7, 8, 9, 18, X and Y, then (ii) measurement of sperm DNA 5mC and 5hmC levels. Results were additionally supported by sperm chromatin integrity assays. The knowledge obtained by implementation of our sequential staining clearly reveals the validity of application of sperm separation methods regarding the chromosomal context of fertilization.

## Materials and methods

### Participants

Biological material consisted of spermatozoa from five healthy normozoospermic volunteers (according to WHO criteria [98]) aged between 25 to 30 years, with a 46,XY karyotype, and without history of reproductive failure. These volunteers were recruited through local advertisement as part of an ongoing research project conducted in the Department of Reproductive Biology and Stem Cells. All men were notified about the purpose of the study and provided written informed consent, according to the Declaration of Helsinki and the approved protocols and guidelines of the Local Bioethical Committee at Poznan University of Medical Sciences (approval no. 669/22).

**Fig. 1:**
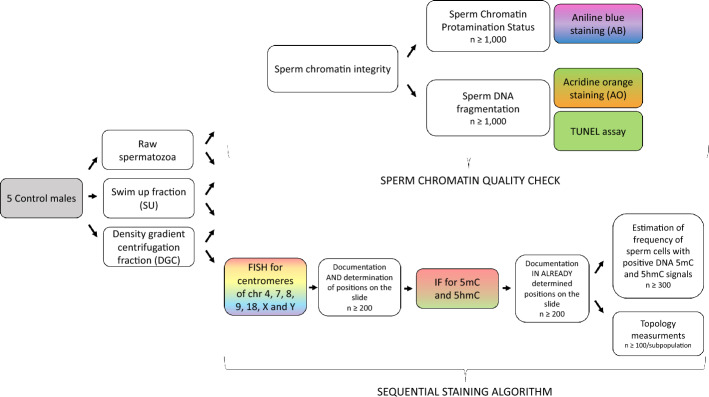
Workflow chart of the experimental approach, showing the step-by-step experimental algorithm included for separation of good-quality fractions, and then two groups of experiments. First (upper panel), a sperm chromatin quality check was done to characterize the group of evaluated cases. Second (lower panel), the sequential staining algorithm involved: (i) determination of chromosomal positioning (centromeres of chromosomes 4, 7, 8, 9, 18, X, and Y) via fluorescence in situ hybridization (FISH), and (ii) subsequent analysis of global DNA methylation (5mC) and hydroxymethylation (5hmC) levels in the same spermatozoa. *n* is the number of spermatozoa measured for each evaluated chromosome

A schematic workflow of the experimental approaches used in this study, including the sperm chromatin quality check and sequential staining algorithm, is presented in Fig. 1.

### Preparation of semen samples

Semen samples were collected via masturbation, after 3–4 days of sexual abstinence. After liquefaction (30 min.) samples were analyzed manually according to the WHO 2021 [98] criteria for semen evaluation (volume, concentration, motility, morphology, and viability) (Table 1). Then, samples were subjected to fractionation via swim up (SU) or Percoll density gradient centrifugation (DGC), fixed, and stored at −20 °C until further use. Also, raw samples (nonfractionated ejaculate samples) were washed out from seminal plasma and spermatozoa were fixed as further described.

**Table 1:** Characteristics of semen parameters of *n* = 5 cases evaluated in the study

Case	Age	Sperm concentration (10^6^/ml)	Ejaculate volume (ml)	Total sperm count (× 10^6^)	Sperm morphology (normal forms; %)	Total motility (%)	Progressive motility (%)
K1	30	101.23	3.0	303.7	21.0	76.0	59.0
K2	26	48.93	2.5	122.3	4.0	74.0	66.0
K3	30	82.00	4.5	369.0	7.0	63.0	55.0
K4	25	80.25	3.8	305.0	4.0	57.0	48.0
K5	30	122.00	2.0	244.0	6.0	67.0	62.0
Mean	28.2	86.88	3.16	268.8	9.5	67.40	58.0
SD	2.49	27.14	1.00	93.07	7.77	7.83	6.89
Lower reference limits for normozoospermia according to WHO guidelines (1)	-	16.00	1.4	39.0	4.0	42.0	30.0

SD, standard deviation

### Swim up selection

Liquefied semen sample (1 ml) was placed in a round-bottomed tube (15 ml; Falcon, Corning, Tamaulipas, Mexico) and centrifuged (8 min, 1800 rpm) with warm (37 °C) F10 medium (cat. no. 134464606, Biomed-Lublin, Poland) to remove seminal plasma, as maintaining physiological temperature preserves sperm viability and reflects the in vivo conditions encountered upon entry into the female reproductive tract, where sperm acquire their highest activity [104]. The supernatant was discarded, and fresh, warm medium (37 °C, 2 ml) was gently overlayered over the pellet. After incubation (37 °C, 45 min), two fractions were separated: an upper layer with motile spermatozoa, and the bottom one with nonmotile spermatozoa. For the target examinations, only the upper layer was used for study purposes, being called the “swim up fraction” (SU). Then, samples were subjected to three rounds of fixation in fresh fixative solution (methanol:acetic acid, 3:1 v/v, −20 °C, POCh, Gliwice, Poland) during 20 min each, and then stored at –20 °C until further use.

### Percoll density gradient centrifugation

A 90%/47% gradient of Percoll (Sigma-Aldrich, St. Louis, MO, USA, P1644) was used for separation of spermatozoa [105]. At the top of the gradient, up to 3 ml of liquefied semen sample was carefully added and centrifuged at 1495 rpm (450g) for 30 min at room temperature (RT). For the purpose of this study, we selected the enriched motile and good morphology spermatozoa contained in the bottom fraction of Percoll gradients, being called for study purposes the “density gradient centrifugation fraction” (DGC). Each sample was washed three times in 1 × PBS (Gibco, Paisley, UK, pH 7.4) at RT to deplete any traces of Percoll solution, followed by three rounds of fixation (methanol:acetic acid, 3:1 v/v, −20 °C) during 20 min each. Fixed samples were stored at –20 °C until further use.

### Sperm chromatin integrity

Sperm chromatin integrity status was evaluated using three tests according to previously published protocols [23, 26, 106–110]. To determine the sperm chromatin protamination level, Aniline Blue (AB) staining was used. To evaluate sperm DNA fragmentation, Acridine Orange (AO) staining was performed for sperm single-stranded DNA (ssDNA) fragmentation level estimation, followed by a terminal deoxynucleotidyl transferase dUTP nick end labeling (TUNEL) assay for determination of sperm double-stranded (dsDNA) and single-stranded DNA (ssDNA) fragmentation level.

### Sperm chromatin protamination

Sperm chromatin protamination status was evaluated by application of Aniline Blue (AB) staining. Briefly, fixed spermatozoa were spread onto microscopic slides, washed in 2× saline sodium citrate (SSC, pH 7.2, W302600; Sigma-Aldrich) for 3 min at RT, and air-dried. Next, 100 µl of 1% eosin-Y solution (Merck, Darmstadt, Germany) was applied on the slides for 3 min at RT, followed by water rinsing. Then, slides were stained in acidic 5% solution of Aniline Blue (cat. no. 95290, Water Blue, Fluka, Darmstadt, Germany) for 5 min at RT, rinsed off with water, and air-dried. The slides were analyzed using a light microscope (Leica DM5500, 100× oil immersion objective; LAS X software; Germany). In each sample, at least *n* ≥ 1000 spermatozoa were evaluated. Aniline Blue is a reagent that binds to lysine residues in histones, resulting in dark-blue staining. Three populations of spermatozoa can be distinguished: pink—spermatozoa with proper protamines to histones ratio, purple—spermatozoa with disturbed protamines to histones ratio (called “semiprotaminated for the purpose of this study) and navy blue—deprotaminated spermatozoa with predominant number of remaining histones.

### Sperm DNA fragmentation (SDF)

Sperm DNA fragmentation status was evaluated using two methods: (i) an Acridine Orange (AO) test to quantify single-stranded (ss) DNA breaks and (ii) a TUNEL assay to estimate the level both of double-stranded (ds) and single-stranded (ss) breaks of DNA. For both tests, spermatozoa were analyzed and documented using fluorescence microscope (Leica DM5500, Germany; 63× oil immersion objective and filters: DAPI, SpG, SpO, triple BGR) and LAS X software (Leica).

An AO test was carried out on slides with fixed spermatozoa, as described previously [106]. Briefly, dry slides were stained with freshly prepared Acridine Orange solution: 10 ml of 1% AO (cat. no. 235474, Sigma-Aldrich) in distilled water added to a mixture of 40 ml 0.1 M citric acid (cat. no. 251275, Sigma–Aldrich) and 2.5 ml 0.3 M Na_2_HPO_4_·7H_2_O (cat. no. S9390, Sigma–Aldrich; pH 2.5). After incubation in the dark (5 min, RT), the slides were rinsed off with distilled water and air-dried. The coverslips were applied onto slides, and in each sample at least *n* ≥ 1000 spermatozoa were evaluated. The Acridine Orange test detects and quantifies DNA ss breaks by utilizing a reagent that binds to DNA and then forms two types of complexes: A and B. The type A complex, as a result of intercalation of monomers between bases in double-stranded DNA, exhibits a maximum absorption at a wavelength of 502 nm (green fluorescence). The type B complex is formed when Acridine Orange molecules aggregate on a single strand of denatured DNA. The maximum absorption for this complex is at 475 nm (red or yellow fluorescence). Thus, two populations of spermatozoa can be distinguished: green—without ssDNA fragmentation, and yellow or red—with ssDNA fragmentation.

The TUNEL assay was carried out according to the manufacturer’s protocol, using the FlowTACS apoptosis detection kit (cat. no. 4817-60-K, R&D Systems, Minneapolis, MN, USA) to identify spermatozoa with fragmented DNA (presence of nicks) by creation of a complex between biotinylated DNA fragments and streptavidin-conjugated fluorescein (FITC) in the presence of terminal deoxynucleotidyl transferase (TdT) [26, 106–108, 110]. Briefly, after permeabilization in 0.1% Triton/sodium citrate solution (15 min, RT), slides were washed with 1× PBS (Gibco, Paisley, UK, pH 7.4), followed by incubation with TdT and a labeling buffer (1 h at 37 °C in the dark). Next, the slides were washed with 1× PBS twice and air-dried. The slides were counterstained with 15 µl of DAPI. TUNEL-positive cells (with fragmented DNA) were fluorescently labeled (green color), then visualized and counted under a fluorescence microscope. In each sample, at least *n* ≥ 1000 spermatozoa were evaluated.

After the quality estimation of semen parameters and sperm chromatin described above, the sequential staining algorithm was implemented, as schematically presented in Fig. 1.

### Fluorescence in situ hybridization (FISH)

Slides with fixed spermatozoa were incubated in a decondensation solution (15 mmol dithiothreitol/ddH_2_O, DTT; cat. no. 111474, Merck KGaA, Darmstadt, Germany) at 43 °C for 7–8 min. A chromatin loosening step is required for hybridization of FISH probes to DNA. Then, the slides were rinsed in 2× SSC (pH 7.2, RT) and air-dried. The volume of the sperm nucleus increased 1.4–1.5-fold, maintaining the shape and geometrical features of the spermatozoon. The FISH technique was prepared according to the manufacturer’s protocol (Cytocell, Cambridge, UK) with minor modifications [23, 109, 111]. Briefly, slides with decondensed spermatozoa were washed in 2× SSC (3 min, RT), dehydrated with ethanol series (70–85–99%), and allowed to dry. To investigate the localization of centromeres of chromosomes 4, 7, 8, and 9, 2.5 µl of each α-satellite or satellite III probe (Cytocell, UK) was applied (Additional file 1). The final mix volume was adjusted to 10.0 µl using hybridization buffer. To investigate the localization of centromeres of chromosomes 18, X, and Y, 7.0 µl of 18 centromere probe, and 2.5 µl of X and Y probes each (Cytocell, UK) were used, with hybridization buffer adjustment to 20 µl (Additional file 1). The slides and the mix of probes were prewarmed at 37°C for 5 min. After that, the probe mixture was placed on a slide and covered with coverslip (24 × 24 mm or 24 × 32 mm), sealed with a rubber glue, and denatured in 75 °C for 2 min. The slides were placed in a humid, lightproof container and incubated overnight at 37 °C for hybridization. Next, the coverslip was removed and the slide was immersed in: 0.4 × SSC (pH 7.0) at 72 °C for 2 min, and 2 × SSC, 0.05% Tween-20 (pH 7.0) at RT for 30 s, to get rid of any remaining unbound probes. Then, DAPI/antifade was applied on each slide and covered with a coverslip (24 × 60 mm). The color was allowed to develop in the dark for 10 min.

FISH results were scored using fluorescence microscope (Leica DM5500, Germany; 63× oil immersion objective, DAPI, SpG, SpO, SpA, triple BGR filters and motorized stage). Image acquisition was performed with LAS X software (Leica, Germany) with Navigator functions that allowed to document the position of each particular sperm on the slide and use the same localization for the next round of staining in the sequential staining algorithm.

### Localization of the centromeres

The linear and radial positioning measurement methods of chromosomes in spermatozoa were developed by Zelenskaya and Zalensky [12]. Owing to the geometrical features of the spermatozoa on the microscopic slide, developed measurement patterns allow the results to be normalized and located within the nuclear space, ensuring consistent and reliable comparisons across samples. Those patterns were explained and successfully used in our previous topology studies [23, 26, 106, 109–116]. In this study, we evaluated the localization of chromosomes 4, 7, 8, 9, 18, X, and Y. The selection of those chromosomes was based on their distinct size and gene density, covering both large (4, X), medium [7–9], and small (18, Y), as well as gene-rich (7, 9, X) and gene-poor chromosomes (18, Y). This selection enables the most representative overview of chromosomal organization. The size of the evaluated chromosomes, the number of genes, and the gene density of selected chromosomes are presented in Additional file 2. In addition, chromosomes X and Y were specifically included as the sex chromosomes, given their particular biological importance, as mentioned in the “Introduction” section. All of the evaluated chromosomes have also been routinely analyzed in our laboratory for years and have been used in previous studies by our research team [23, 26, 106, 109–116]. Additionally, only morphologically normal spermatozoa were analyzed in this study, reflecting the selection criteria used in IVF clinics.

### Linear positioning of the centromeres of chromosomes

**Fig. 2:**
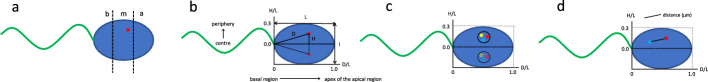
Schematic representation of measurement approaches of centromere localization (red, yellow, and light-blue points) within the sperm nucleus (blue background). **a** Linear positioning: the frequency of FISH signals across three equally divided regions of the nucleus, segmented along its longitudinal “L” axis (“b”, near the basal region; “m”, middle; “a”, near the apical region). **b** Radial positioning: shown as central (deep inside the nucleus, values close to 0.0) or peripheral (near the nuclear membrane, values close to 0.3) localization of the signal, based on normalized D/L (OX axis) and H/L (OY axis) values; D and H are the distances from the FISH signal (red point) to the basal attachment point and the longitudinal axis, respectively; L and l are the longitudinal and short axes, respectively. Dotted lines show mirror images of centromere positioning (the spermatozoa has the property that it can only take two positions on the microscope slide, which are mirror images of each other). **c** Schematically marked chromocenter region (clusters of centromeres). **d** The distance (μm) between pair of centromeres within the sperm nucleus. The idea of radial positioning was first introduced by Zalenskaya and Zalensky [12]

The linear positioning demonstrates the frequency of FISH signals in three equal territories of the sperm nucleus determined along its longitudinal axis: “a”, near the apical region; “m”, middle region, and “b”, near the basal region (related to the tail attachment area) (Fig. 2a).

### Radial positioning of the centromeres of chromosomes

To determine the radial position of chromosomes 4, 7, 8, 9, 18, X, and Y in spermatozoa, the following measurements of the sperm nucleus dimensions were performed: L, the length of the longitudinal axis (from the basal attachment point of the sperm tail to the apex of the apical region); l, the length of the short axis (in the widest part of the nucleus); D, the distance from the FISH signal to the basal attachment point; H, the distance from the FISH signal to the longitudinal axis; and the ratio L/l, the ellipsoidal shape indicating the decondensation ratio of the nucleus (not exceeding 1.4–1.5-fold). The D/L value was used to determine the central localization, towards the “basal–apical” direction, with a maximum value of 1.0. The H/L value defines the localization of the signal in relation to its depth (“centre–periphery” criterion, the proximity to the internal sperm nuclear membrane), with a maximum value of 0.3 (reaching the most peripheral region). The measurement system allows one to depict centromeres’ positions in a coordinate system as the mean D/L ± SE for the OX axis and H/L ± SE for the OY axis, which allows the results to be normalized and located in the nuclear space (Fig. 2b). This kind of normalization enables positioning of signals in a nuclear space that approximates a 3D model, where the relationship between the central and peripheral regions is effectively visualized [12, 109]. This spatial representation can visualize centromere localization both deeply in the center of the sperm nucleus or near the nuclear membrane. For each evaluated case, each chromosome, and for each 5mC and 5hmC groups, at least *n* ≥ 100 FISH signals were measured, which resulted in the analysis of 14,120 spermatozoa, in total. The clusters of intranuclear positions of the analyzed centromeres are presented as specific regions within the sperm nucleus, referred to as chromocenters (Fig. 2c).

### Distances between the centromeres of chromosomes

For each sample, the distance between the centromeres of two chromosomes was measured for at least *n* ≥ 100 spermatozoa. The measurement of the distance between centromeres was performed for the following pairs of chromosomes: chromosomes 4 versus 8, chromosomes 7 versus 9, and chromosomes 18 versus X or Y (Fig. 2d).

### Sperm DNA 5mC and 5hmC levels

On slides with previously defined spermatozoal position and documented topology of centromeres, immunofluorescence (IF) staining for determination of the global sperm DNA methylation (5mC) and hydroxymethylation (5hmC) levels was performed. This method has been validated previously when correlated to thin-layer chromatography (TLC) results and used with further success [107, 108]. Specific antibodies conjugated to fluorochromes and diluted in 1% BSA/1× PBST were used: primary antibodies of mouse anti-5mC 1:200 (clone 33D3, cat. no. MABE146, Merck) and rat anti-5hmC 1:1000 (cat. no. ab106918, Abcam), and secondary antibodies of goat anti-mouse-FITC 1:400 (cat. no. F2012, Sigma-Aldrich) and goat anti-rat-AF594 1:800 (cat. no. ab150160, Abcam). First, samples with fixed sperm smears after FISH staining and documentation were incubated in 1× PBST (pH 7.4) for 10 min at RT, followed by four washes in 1× PBST (pH 7.4, 5 min each, RT). Then, slides were incubated in 25mM DTT/1M Tris–HCl (pH 9.5, 10 min, RT) to slightly decondense the chromatin, followed by two washes in 1× PBST (pH 7.4, 5 min each, RT). To denature the samples, slides were then incubated in 6 N HCl (15 min, RT), 1M Tris–HCl (pH 9.5, 15 min, RT), and 1 × PBST (pH 7.4, 5 min, RT). Next, slides were blocked with 1% BSA/1× PBST for 1 h at RT, and incubated overnight in a humidified container at 4 °C with a mix of primary antibodies. After incubation and a series of rinsing in 1 × PBST (threefold, 5 min each, RT), secondary antibodies conjugated to selected fluorochromes were applied for 1 h at RT. Next, samples were washed twice in 1× PBST (pH 7.4, 5 min each, RT), and DAPI/antifade solution (Vectashield, cat. no. H-1000, Vector Laboratories, Newark, CA, USA) was applied on each slide and covered with a coverslip (24 × 60 mm). The colors were allowed to develop in the dark for 10 min. Results were analyzed using fluorescence microscope (Leica DM5500, Germany; 63× oil immersion objective, DAPI, SpG, SpO, triple BGR, and motorized stage). The acquired images were analyzed using and LAS X software and Navigator options (Leica, Germany).

As mentioned above, spermatozoa were collected in three groups: raw, SU, and DGC sperm fractions. For each fraction and each chromosome, the position on the slide of at least *n* = 200 spermatozoa was documented. Then, a second round of staining for the same sperm with fixed positions (on slides) was performed for estimation of global 5mC and 5hmC levels of sperm DNA. Topology measurement for each case was performed in a total of approximately *n* = 4200 spermatozoa (7 chromosomes × 3 fractions × 5mC/5hmC hyper/hypo states × at least 100 signals each time) (Figs. 1 and 2). Quantitative IF analysis showed that spermatozoa exhibiting high levels of 5mC also tended to display increased 5hmC IF intensity, indicating a correlation between these two DNA modifications within the same cells [117]. Therefore, we focused our topology measurements on spermatozoa with either high or low levels of both epimarks. General results including the whole sperm population were also evaluated.

### Statistical analysis

For verification of the normal distribution of the measurements, the Shapiro–Wilk test was performed. For statistical analysis of sperm chromatin integrity, linear results, and estimation of the 5mC and 5hmC levels, the Mann–Whitney test or unpaired *t*-test with Welch’s correction were applied with a significance level of *α* = 0.05. For statistical analysis of the radial results and distances between the centromeres of chromosomes, Kruskal–Wallis test with Dunn’s multiple comparisons test was carried out at a significance level of *α* = 0.05. All the tests were performed using GraphPad Prism (version 8.4.3) software. Estimation of the common aggregation of centromeres was performed using Ward cluster analysis and visualized as hierarchical trees with linkage to Euclidean distances, utilizing the tool available on the website https://datatab.net/statistics-calculator/cluster.

## Results

The results are presented in two sections: first, the assessment of sperm quality parameters, including chromatin protamination status, sperm DNA fragmentation (SDF), and global DNA methylation and hydroxymethylation levels (5mC and 5hmC), followed by the analysis of chromosomal positioning and epigenetic modifications performed by sequential staining and evaluation of the same individual sperm.

### Sperm chromatin integrity

**Fig. 3:**
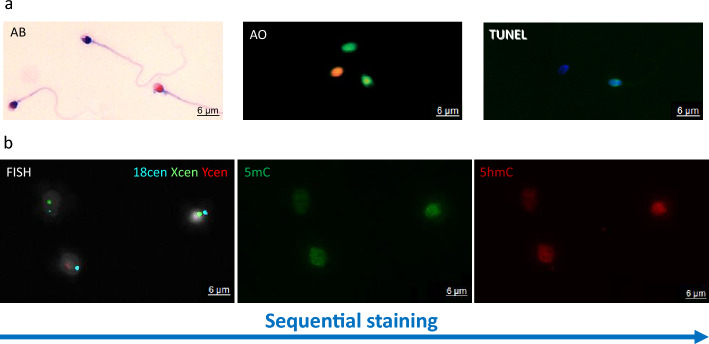
Examples of staining results. **a** Sperm chromatin quality check: (i) Aniline Blue (AB) staining: three different sperm subpopulations: pink with proper chromatin protamination level, purple with disturbed protamination, and navy blue with highly deprotaminated chromatin, (ii) Acridine Orange (AO) staining: two different sperm subpopulations: green without ssDNA fragmentation and orange with ssDNA fragmentation, (iii) TUNEL assay: two sperm nuclei subpopulations: TUNEL-negative (without dsDNA and ssDNA fragmentation) and TUNEL-positive bright-green spermatozoa (with dsDNA and ssDNA fragmentation). **b** Step-by-step sequential staining algorithm of spermatozoa. First—fluorescence in situ hybridization (FISH) for centromeres of selected chromosomes (left panel); second—immunofluorescent staining (IF) for evaluation of global levels of sperm DNA methylation (5mC) and hydroxymethylation (5hmC) (middle and right panels). Images were acquired with light (AB) or fluorescent (AO, TUNEL, FISH, IF) microscope (Leica DM5500, oil immerse objective 63× and SpO/FITC/SpAq/DAPI filters)

**Fig. 4:**
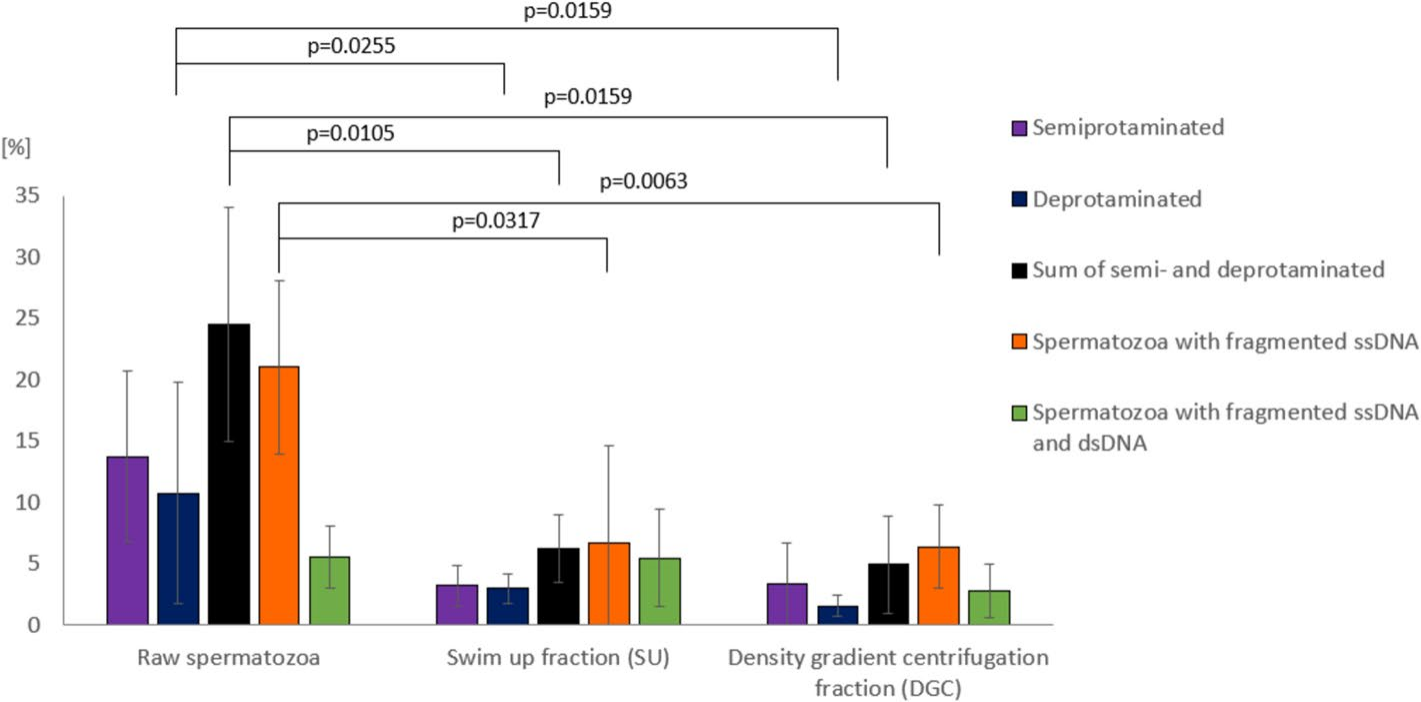
Sperm chromatin integrity results. Deprotamination level—Aniline Blue (AB) staining; DNA fragmentation: ssDNA fragmentation—Acridine Orange (AO) staining, ssDNA and dsDNA fragmentation—TUNEL assay. Bars represent mean values ± SD, dots represent all cases (K1–K5) with measured values. Statistical significance was considered at *p* < 0.05

The results of the sperm chromatin integrity evaluation consisted of the sperm chromatin protamination test (AB) and SDF assays (AO and TUNEL), as presented in Table 2, Figs. 3a and 4, and Additional file 3.

**Table 2:** Results of sperm chromatin integrity evaluation in raw spermatozoa population and good-quality fractions (swim up fraction (SU) and density gradient centrifugation fraction (DGC))

		Raw spermatozoa, mean ± *SD*	SU fraction Mean ± *SD*	DGC fraction Mean ± *SD*
Sperm chromatin protamination [%; Aniline Blue assay, AB]	Properly protaminated (pink)	75.47 ± *9.51*	93.76* ± *2.75*	95.04* ± *3.98*
	Semiprotaminated (purple)	13.77 ± *6.95*	3.22* ± *1.69*	3.39* ± *3.29*
	Deprotaminated (navy blue)	10.76 ± *9.03*	3.02 ± *1.22*	1.57 ± *0.84*
	Sum of semi- and deprotaminated	24.53 ± *9.51*	6.24* ± *2.75*	4.96* ± *3.97*
Spermatozoa with fragmented DNA [%]	Spermatozoa with fragmented ssDNA [Acridine Orange staining, AO]	21.03 ± *7.04*	6.70* ± *7.96*	6.39** ± *3.40*
	Spermatozoa with fragmented ssDNA and dsDNA [TUNEL assay]	5.54 ± *2.53*	5.50 ± *3.94*	2.76 ± *2.18*

^*^ Values statistically significant (*p* < 0.05) with respect to the raw spermatozoa values

^**^ Values highly statistically significant (*p* < 0.01) with respect to the raw spermatozoa values

The results of AB staining showed that the average level of sperm DNA protamination was 75.47 ± 9.51% in raw spermatozoa, 93.76 ± 2.75% in SU spermatozoa, and 95.04 ± 3.98% in DGC spermatozoa. Thus, the average level of sperm DNA protamination significantly increased, in comparison with the raw fraction, by 1.24-fold (+ 24.24%) for SU spermatozoa (*p* = 0.0105) and 1.3-fold (+ 25.93%) for DGC spermatozoa (*p* = 0.0159) (Table 2 and Fig. 4).

Results of AO staining showed that the mean frequency of sperm with ssDNA fragmentation was 21.03 ± 7.04% in raw spermatozoa, 6.70 ± 3.94% in SU spermatozoa, and 6.39 ± 2.18% in DGC spermatozoa. Thus, the mean frequency of spermatozoa with ssDNA fragmentation significantly decreased, by 3.14-fold (−68.14%) in SU spermatozoa (*p* = 0.0317) and 3.29-fold (−69.6%) in DGC spermatozoa (*p* = 0.0063) (Table 2 and Fig. 4).

TUNEL assay evaluation revealed that the mean frequency of sperm with dsDNA and ssDNA fragmentation was 5.54 ± 2.53% in raw spermatozoa, 5.5 ± 3.94% in SU spermatozoa, and 2.76 ± 2.18% in DGC spermatozoa. However, no statistically significant differences between sperm fractions were found (Table 2 and Fig. 4).

### Sperm DNA 5mC and 5hmC levels

The mean frequency of spermatozoa with highly methylated DNA (5mC) and hydroxymethylated DNA (5hmC), estimated from the raw, SU, and DGC fractions, are presented in Table 3.

**Table 3:** Mean global sperm DNA methylation (5mC) and hydroxymethylation (5hmC) status estimated for spermatozoa from raw spermatozoa and good-quality fractions (swim up fraction (SU) and density gradient centrifugation fraction (DGC))

Fraction	Frequency of spermatozoa with positive signal of DNA methylation (5mC, %)	*p*-Value (versus raw spermatozoa)	Frequency of spermatozoa with positive signal of DNA hydroxymethylation (5hmC, %)	*p*-Value (versus raw spermatozoa)
Raw spermatozoa	57.82 ± 6.46 (45.48–68.95)	–	57.86 ± 6.46 (45.48–68.95)	–
SU fraction	64.87 ± 6.57 (54.26–75.41)	0.0062	64.61 ± 6.46 (54.26–75.13)	0.0079
DGC fraction	61.59 ± 5.24 (49.24–69.06)	ns	61.79 ± 5.25 (49.24–69.62)	ns

ns, not significant

The analysis revealed that the levels of the mean 5mC or 5hmC frequencies were 57.82 ± 6.456% or 57.86 ± 6.460% (5mC or 5hmC, respectively) in raw spermatozoa (Table 3). In contrast, SU spermatozoa exhibited a significantly higher level of 5mC or 5hmC (+12.19% or +11.66%, respectively) when compared with raw spermatozoa, averaging 64.87 ± 6.572% (*p* = 0.0062) or 64.61 ± 6.456% (*p* = 0.0079), respectively (Table 3). On the other hand, no statistically significant differences were observed between DGC spermatozoa and raw spermatozoa, even if the average 5mC or 5hmC frequencies were slightly increased: 61.59 ± 5.241% (+6.52%) or 61.79 ± 5.254% (+6.79%), respectively (Table 3).

### Sperm FISH analysis—chromosomal positioning

In this study, we determined chromosomal positioning within the spermatozoa nucleus through sequential stainings performed for the same individual sperm (cell by cell, in situ on a microscopic slide, as indicated in Figs. 1, 2, and 3b). First, FISH was performed on spermatozoa from the raw, SU, and DGC fractions, followed by documentation of spermatozoa position on microscopic slides. Second, IF staining for 5mC and 5hmC was applied on the same slides, and subsequent topology measurements were performed. This allowed us to establish the chromosomal positions in not only nondifferentiated spermatozoa populations, but also with low (hypo-5mC−/5hmC−) and high (hyper-5mC+/5hmC+) levels of DNA methylation/hydroxymethylation.

### Linear positioning

**Fig. 5:**
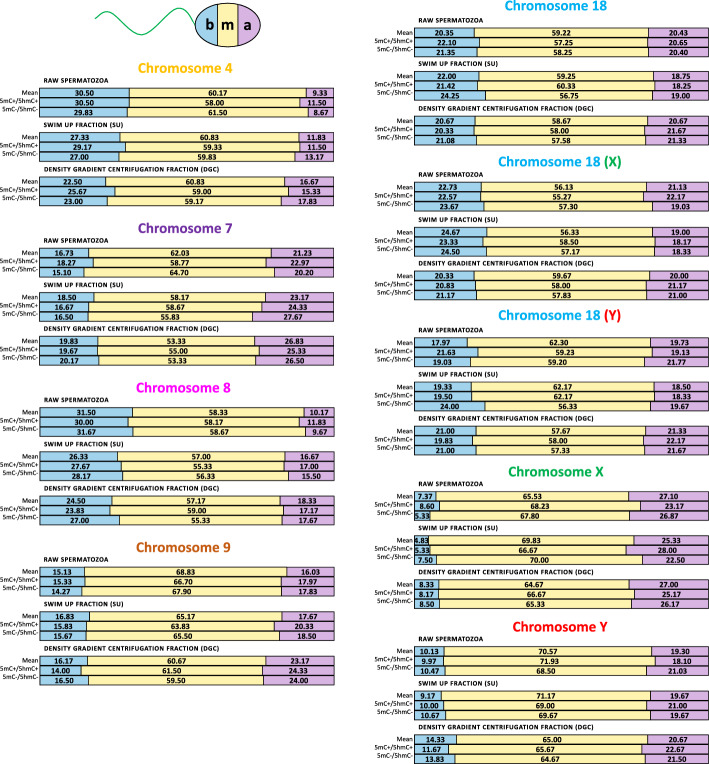
Linear positioning of centromeres within the sperm nucleus. Comparison of frequencies of chromosomes 4, 7, 8, 9, 18, X, and Y in particular regions of the spermatozoa nucleus (b, near the basal region; m, middle part; a, near the apical region) in raw spermatozoa, swim-up fraction (SU), and density gradient centrifugation fraction (DGC), including also differentiation into high- (5mC/5hmC+) and low-methylated/hydroxymethylated (5mC/5hmC−) sperm populations (according to Table 4)

The linear positioning consisted of frequency values of each chromosome in three regions of the sperm nucleus as presented in Table 4 and Fig. 5 (mean values), supported by Additional file 4 (data for individual cases K1–K5).

**Table 4:** Linear positioning of centromeres of chromosomes 4, 7, 8, 9, 18, X, and Y in human spermatozoa of *n* = 5 male normozoospermic cases (K1–K5)

Chromosome		a, near the apical region; m, middle; b, near the basal region	Raw spermatozoa		Raw spermatozoa 5mC+/5hmC+		Raw spermatozoa 5mC−/5hmC−		SU fraction		SU fraction 5mC+/5hmC+		SU fraction 5mC−/5hmC−		DGC fraction		DGC fraction 5mC+/5hmC+		DGC fraction 5mC−/5hmC−	
			Mean [%]	SD	Mean [%]	SD	Mean [%]	SD	Mean [%]	SD	Mean [%]	SD	Mean [%]	SD	Mean [%]	SD	Mean [%]	SD	Mean [%]	SD
4		a	9.33	2.97	11.50	3.08	8.67	4.99	11.83	5.76	11.50	4.54	13.17	7.39	16.67	8.64	15.33	6.50	17.83	8.14
		m	60.17	4.38	58.00	3.80	61.50	5.05	60.83	13.04	59.33	11.43	59.83	12.46	60.83	11.61	59.00	8.28	59.17	11.62
		b	30.50	5.45	30.50	3.21	29.83	3.41	27.33	10.41	29.17	7.86	27.00	8.01	22.50	4.82	25.67	2.73	23.00	4.02
7		a	21.23	2.63	22.97	0.90	20.20	5.78	23.17	9.60	24.33	10.43	27.67	8.85	26.83	7.06	25.33	6.25	26.50	3.79
		m	62.03	3.19	58.77	3.50	64.70	7.05	58.17	14.76	58.67	13.90	55.83	12.49	53.33	9.20	55.00	7.55	53.33	8.23
		b	16.73	2.50	18.27	3.21	15.10	3.97	18.50	5.51	16.67	3.63	16.50	4.54	19.83	4.31	19.67	3.89	20.17	5.96
8		a	10.17	4.06	11.83	2.97	9.67	2.67	16.67	7.57	17.00	6.68	15.50	6.84	18.33	9.05	17.17	9.80	17.67	11.08
		m	58.33	5.59	58.17	2.53	58.67	2.33	57.00	15.09	55.33	11.76	56.33	13.42	57.17	11.06	59.00	11.58	55.33	7.70
		b	31.50	7.46	30.00	4.89	31.67	2.12	26.33	7.81	27.67	5.69	28.17	8.47	24.50	3.04	23.83	4.39	27.00	5.67
9		a	16.03	6.70	17.97	4.27	17.83	4.74	17.67	6.70	20.33	9.73	18.50	6.75	23.17	10.66	24.33	10.86	24.00	9.27
		m	68.83	8.07	66.70	7.17	67.90	6.05	65.17	13.46	63.83	15.52	65.50	8.83	60.67	12.51	61.50	12.34	59.50	13.39
		b	15.13	4.03	15.33	3.98	14.27	2.88	16.83	6.91	15.83	6.69	15.67	3.25	16.17	3.15	14.00	2.16	16.50	5.05
18	Unselected	a	20.43	4.43	20.65	3.59	20.40	6.38	18.75	6.99	18.25	7.10	19.00	7.55	20.67	4.86	21.67	6.65	21.33	5.36
		m	59.22	4.13	57.25	6.30	58.25	3.99	59.25	12.21	60.33	9.42	56.75	14.24	58.67	7.78	58.00	7.93	57.58	8.10
		b	20.35	4.83	22.10	4.75	21.35	5.70	22.00	6.79	21.42	4.45	24.25	8.03	20.67	3.54	20.33	2.67	21.08	4.28
	18-X	a	21.13	5.83	22.17	4.40	19.03	6.27	19.00	7.87	18.17	8.55	18.33	7.05	20.00	5.74	21.17	7.18	21.00	5.76
		m	56.13	7.82	55.27	5.42	57.30	6.82	56.33	10.83	58.50	8.24	57.17	15.00	59.67	7.85	58.00	8.91	57.83	9.57
		b	22.73	7.12	22.57	3.91	23.67	6.47	24.67	5.67	23.33	3.06	24.50	10.15	20.33	2.54	20.83	4.37	21.17	4.99
	18-Y	a	19.73	4.11	19.13	3.62	21.77	6.95	18.50	7.30	18.33	6.54	19.67	8.83	21.33	4.84	22.17	6.63	21.67	6.12
		m	62.30	2.95	59.23	7.26	59.20	3.53	62.17	13.69	62.17	10.79	56.33	14.39	57.67	8.13	58.00	7.06	57.33	8.02
		b	17.97	3.01	21.63	6.40	19.03	5.70	19.33	8.85	19.50	8.22	24.00	7.13	21.00	4.84	19.83	2.31	21.00	4.39
X		a	27.10	11.28	23.17	8.92	26.87	10.44	25.33	8.85	28.00	14.10	22.50	7.55	27.00	11.27	25.17	11.92	26.17	9.80
		m	65.53	10.00	68.23	9.42	67.80	9.65	69.83	10.09	66.67	16.08	70.00	10.56	64.67	14.03	66.67	12.35	65.33	13.01
		b	7.37	2.35	8.60	1.47	5.33	1.76	4.83	2.91	5.33	3.75	7.50	4.37	8.33	5.80	8.17	4.84	8.50	6.05
Y		a	19.30	6.60	18.10	9.71	21.03	9.17	19.67	7.23	21.00	11.20	19.67	7.96	20.67	10.63	22.67	12.21	21.50	13.29
		m	70.57	3.13	71.93	5.03	68.50	4.80	71.17	12.55	69.00	15.58	69.67	13.83	65.00	14.35	65.67	13.87	64.67	17.40
		b	10.13	5.82	9.97	5.30	10.47	5.23	9.17	7.24	10.00	6.10	10.67	6.98	14.33	4.73	11.67	2.57	13.83	5.09

For each of the evaluated autosomes, the position of centromeres was defined for unselected spermatozoa according to sex chromosomes. For chromosome 18, additional measurements were done according to X- or Y-bearing spermatozoa. At least *n* = 100 signals were evaluated for each case and each chromosome. SU, swim up fraction; DGC, density gradient centrifugation fraction; 5mC, global sperm DNA methylation; 5hmC, global sperm DNA hydroxymethylation

Regardless of the fraction and the level of 5mC/5hmC, most chromosomes preferentially localized in the middle (“m”) part of the sperm nucleus. The X chromosome was predominantly found near at the apical nuclear region (“a”), and chromosomes 4 and 8 were mostly localized at the basal nuclear region (“b”). In sperm fractions, as well as in sperm with differentially methylated and hydroxymethylated DNA, there were no statistically significant differences in the localization of all evaluated chromosomes. However, the greatest difference in linear positioning was observed for chromosome 4, between raw and DGC spermatozoa, where the centromeres were shifted toward the apical nuclear region (“a”: 9.33 ± 2.97% and 16.67 ± 8.64%, respectively; “m”: 60.17 ± 4.38% and 60.83 ± 11.61%, respectively; “b”: 30.50 ± 5.45% and 22.50 ± 4.82%, respectively) (Table 4).

To make the text clear and compact, we present the description of the linear topology in spermatozoa with various levels of DNA methylation and hydroxymethylation in the following order: name of fraction, fraction highly methylated or hydroxymethylated DNA (described as: fraction 5mC+/5hmC+), fraction low-methylated or -hydroxymethylated DNA (described as: fraction 5mC−/5hmC−).

### Raw spermatozoa

All the evaluated chromosomes in raw spermatozoa, raw spermatozoa 5mC+/5hmC,+ or raw spermatozoa 5mC−/5hmC− DNA were preferentially localized in the middle nuclear region (“m”) (62.57 ± 4.86%, 61.51 ± 5.85%, or 62.65 ± 4.60%, respectively). At the apical nuclear region (“a”), the mean frequency of centromeres observed was 18.27 ± 5.62%, 18.61 ± 4.39%, or 18.39 ± 5.80%, respectively, with the most apical position noted for the centromere of chromosome X (27.10 ± 11.28%, 23.17 ± 8.92%, or 26.87 ± 10.44%, respectively). The mean localization near the basal region (“b”) was 19.16 ± 8.21%, 19.89 ± 7.74%, or 18.97 ± 8.68%, respectively, and was preferentially exhibited by the centromere of chromosome 4 (mean 30.50 ± 5.45%, 30.50 ± 3.21%, or 29.83 ± 3.41%, respectively) and chromosome 8 (31.50 ± 7.46%, 30.00 ± 4.89%, or 31.67 ± 2.12%, respectively) (Table 4).

### Swim up fraction (SU)

For SU spermatozoa, in SU 5mC+/5hmC+ or in SU 5mC−/5hmC−, the analyszd chromosomes also predominantly occupied the middle part of the sperm nucleus (“m”), with a mean frequency of 62.21 ± 5.43%, 61.54 ± 4.33%, or 60.82 ± 5.92%, respectively. At the apical nuclear region (“a”), the centromeres frequencies were 18.95 ± 3.82%, 19.66 ± 4.65%, or 19.33 ± 4.10%, with chromosome X displaying the most apical localization in SU spermatozoa and in SU 5mC+/5hmC+ (25.33 ± 8.85% and 28.00 ± 14.10%, respectively) and chromosome 7 being the most apically positioned in SU 5mC−/5hmC− (27.67 ± 8.85%). Meanwhile, centromeres positioned at the basal region (“b”) accounted for 18.78 ± 7.63%, 18.77 ± 7.80%, or 19.81 ± 7.45%, respectively, with chromosomes 4 (27.33 ± 10.41%, 29.17 ± 7.86%, or 27.00 ± 8.01%, respectively) and 8 (26.33 ± 7.81%, 27.67 ± 5.69% or 28.17 ± 8.47%, respectively) being the most frequently localized in this area (Table 4).

### Percoll density gradient centrifugation fraction (DGC)

For DGC spermatozoa, in DGC 5mC+/5hmC+, or in DGC 5mC−/5hmC−, most of the analyzed chromosomes were also found in the middle nuclear region (“m”), with a mean frequency of 59.74 ± 3.66%, 60.09 ± 3.84%, or 58.90 ± 3.94%, respectively. Centromeres positioned at the apical nuclear region (“a”) were detected with a mean frequency of 21.63 ± 3.51%, 21.67 ± 3.44%, or 21.96 ± 8.07%, respectively, with the most apical localization found for chromosome X (27.00 ± 11.27%) in DGC spermatozoa, and chromosome 7 in DGC 5mC+/5hmC+levels, and in DGC 5mC−/5hmC− (25.33 ± 6.25% and 26.50 ± 3.79%, respectively). Meanwhile, centromeres located at the basal nuclear region (“b”) were detected with a mean frequency of 18.63 ± 4.92%, 18.22 ± 5.75%, or 19.14 ± 5.45%, with chromosomes 4 (22.50 ± 4.82%, 25.67 ± 2.73%, or 23.00 ± 4.02%, respectively) and 8 (24.50 ± 3.04%, 23.83 ± 4.39%, or 27.00 ± 5.67%, respectively) predominantly found in this area (Table 4).

### Radial positioning

**Fig. 6:**
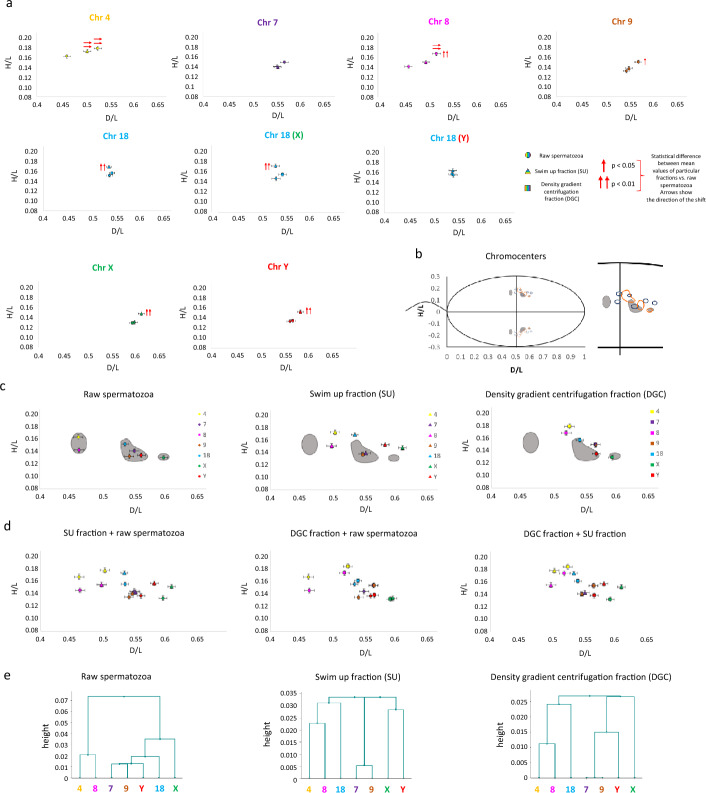
Radial positioning of the centromeres of examined chromosomes (4, 7, 8, 9, 18, X, and Y) within the human spermatozoon nucleus. Three populations of spermatozoa were evaluated in *n* = 5 normozoospermic cases (K1–K5): raw spermatozoa, swim up fraction (SU), and density gradient centrifugation fraction (DGC). Positioning of the centromeres was determined as the points on coordinate system: D/L ± SE (OX axis) and H/L ± SE (OY axis) (according to data in Table 5). **a** Positioning results for individual centromeres (circle: raw spermatozoa; triangle: swim up fraction (SU); square: density gradient centrifugation fraction (DGC)). Localizations that differ significantly from the raw spermatozoa are indicated by arrows: double arrow for *p* < 0.01, single arrow for *p* < 0.05. Arrows also indicate the direction of the observed shift/repositioning of a centromere. Bars show standard errors (SE). **b** Schematic region within the sperm nucleus occupied by chromocenters (solid lines), along with the investigated centromeres. Dotted lines represent their mirror images: raw spermatozoa (gray areas), swim-up fraction (SU; dark blue), and density gradient centrifugation fraction (DGC, orange). **c** The chromocenter areas represented for each sperm population, with grey color for raw spermatozoa as a background for visualization of shifts when compared with swim up (SU) and density gradient centrifugation (DGC) fractions. **d** Schematic diagrams showing chromosome distributions for raw spermatozoa versus SU fraction, raw spermatozoa versus DGC fraction, and SU fraction versus DGC fraction comparisons. **e** Positions of analyzed centromeres represented by hierarchical Ward clustering analysis showing the reciprocal positions of selected chromosomal pairs

The radial positioning results are presented in Tables 5 and 6, Fig. 6, and Additional files 5–7.

**Table 5:** Radial positioning of centromeres of chromosomes 4, 7, 8, 9, 18, X, and Y in human spermatozoa of *n* = 5 male normozoospermic cases (K1–K5)

Chromosome		Raw spermatozoa	Raw spermatozoa 5mC+/5hmC+	Raw spermatozoa 5mC−/5hmC−	SU fraction	SU fraction 5mC+/5hmC+	SU fraction 5mC−/5hmC−	DGC fraction	DGC fraction 5mC+/5hmC+	DGC fraction 5mC−/5hmC−
4	D/L	0.462	0.465	0.465	0.504→→	0.495	0.503→→	0.525→→	0.513→→	0.517→→
	SE	0.008	0.008	0.008	0.007	0.008	0.008	0.008	0.008	0.008
	H/L	0.163	0.162	0.163	0.173	0.173	0.170	0.179	0.176	0.169
	SE	0.004	0.004	0.004	0.004	0.004	0.004	0.004	0.004	0.004
7	D/L	0.552	0.556	0.558	0.553	0.561	0.567	0.567	0.566	0.562
	SE	0.008	0.008	0.008	0.007	0.007	0.007	0.008	0.008	0.008
	H/L	0.141	0.149	0.141	0.140	0.143	0.144	0.150	0.150	0.149
	SE	0.004	0.004	0.004	0.003	0.003	0.003	0.004	0.004	0.004
8	D/L	0.463	0.479	0.464	0.498	0.497	0.493	0.520→→	0.508→→	0.507→→
	SE	0.008	0.008	0.008	0.008	0.008	0.008	0.008	0.008	0.008
	H/L	0.142	0.156	0.136	0.151	0.150	0.159↑	0.169↑↑	0.162↑↑	0.157
	SE	0.004	0.004	0.004	0.003	0.004	0.004	0.004	0.004	0.004
9	D/L	0.543	0.552	0.550	0.548	0.556	0.548	0.567	0.574	0.570
	SE	0.007	0.007	0.007	0.007	0.007	0.007	0.007	0.007	0.007
	H/L	0.132	0.143	0.136	0.138	0.144	0.139	0.150↑	0.149↑	0.146
	SE	0.004	0.004	0.003	0.003	0.003	0.003	0.004	0.004	0.004

Chromosome			Raw spermatozoa	Raw spermatozoa 5mC+/5hmC+	Raw spermatozoa 5mC−/5hmC−	SU fraction	SU fraction 5mC+/5hmC+	SU fraction 5mC−/5hmC−	DGC fraction	DGC fraction 5mC+/5hmC+	DGC fraction 5mC−/5hmC−
18	Unselected	D/L	0.536	0.533	0.537	0.535	0.532	0.536	0.541	0.545	0.539
		SE	0.006	0.006	0.006	0.006	0.005	0.006	0.005	0.006	0.005
		H/L	0.152	0.151	0.151	0.169↑↑	0.170↑↑	0.169↑↑	0.157^a^	0.156	0.160
		SE	0.003	0.003	0.003	0.003	0.002	0.003	0.002	0.002	0.003
	18-X	D/L	0.531	0.536	0.530	0.530	0.525	0.536	0.544	0.547	0.542
		SE	0.008	0.009	0.008	0.008	0.008	0.008	0.008	0.008	0.008
		H/L	0.148	0.155	0.149	0.173↑↑	0.172↑↑	0.171↑↑	0.156^a^	0.157	0.160
		SE	0.004	0.004	0.004	0.004	0.003	0.004	0.004	0.003	0.004
	18-Y	D/L	0.542	0.530	0.544	0.541	0.539	0.535	0.539	0.544	0.535
		SE	0.008	0.008	0.008	0.008	0.008	0.008	0.008	0.008	0.008
		H/L	0.156	0.147	0.153	0.165	0.169	0.167	0.157	0.154	0.160
		SE	0.004	0.004	0.004	0.004	0.004	0.004	0.003	0.003	0.004
X		D/L	0.598	0.585	0.605	0.612	0.617	0.606	0.594	0.595	0.592
		SE	0.007	0.007	0.006	0.006	0.006	0.006	0.006	0.006	0.006
		H/L	0.130	0.124	0.134	0.148↑↑	0.147↑↑	0.153↑↑	0.129	0.133	0.134
		SE	0.004	0.003	0.004	0.003	0.003	0.003	0.003	0.003	0.003
Y		D/L	0.563	0.562	0.573	0.584	0.587	0.577	0.568	0.573	0.565
		SE	0.006	0.006	0.007	0.006	0.006	0.007	0.007	0.006	0.007
		H/L	0.134	0.132	0.136	0.153↑↑	0.154↑↑	0.148	0.135^b^	0.142	0.134^b^
		SE	0.004	0.004	0.004	0.003	0.003	0.003	0.003	0.003	0.003

For each of the evaluated autosomes, the position of centromeres was defined for unselected spermatozoa according to sex chromosomes. For chromosome 18, additional measurements were done according to X- or Y-bearing spermatozoa. At least *n* = 100 signals were evaluated for each case and each chromosome. SU, swim up fraction; DGC, density gradient centrifugation fraction; 5mC, global sperm DNA methylation; 5hmC, global sperm DNA hydroxymethylation; D/L, signal distance along the tail–acrosome axis; H/L, signal proximity to the nuclear periphery (according to Fig. 2); SE, standard error on the mean

Mean values statistically significant (*p* < 0.05) with respect to the mean values of raw spermatozoa are marked with arrows:

↑ centromere localized closer to the periphery of the nucleus

↓ centromere localized closer to the center of the nucleus

→ centromere localized closer to the apical area

← centromere localized closer to the basal area

Mean values with high statistical significance (*p* < 0.01) with respect to the mean values of raw spermatozoa are marked with double arrows:

↑↑ centromere localized closer to the periphery of the nucleus

↓↓ centromere localized closer to the center of the nucleus

→ → centromere localized closer to the apical area

← ← centromere localized closer to the basal area

Mean values with statistical significance between good-quality fractions are marked with letters:

^a^ Significantly different from SU fraction (*p* < 0.05), centromere localized closer to the center of the nucleus

^b^ Highly significantly different from SU fraction (*p* < 0.01), centromere localized closer to the center of the nucleus

**Table 6:** Summary of repositioned centromeres among evaluated chromosomes between fractions

	Direction of repositioning			
	Basal–apical	Center–periphery	Both	None
SU fraction vs. Raw spermatozoa	4	18 X Y		7 8 9
DGC fraction versus raw spermatozoa	4	9	8	7 18 X Y
SU fraction versus DGC fraction		18 X Y		4 7 8 9

SU, swim up fraction; DGC, density gradient centrifugation fraction. “Basal–apical” means that centromere was shifted from the basal to the apical region. “Center–periphery” means repositioning from central area toward the nuclear periphery. “Both” stands for centromere repositioning observed in both directions: tail–acrosome and center–periphery, while “None” stands for no statistically significant change in centromere localization between fractions

The spatial positioning of all the studied centromeres in raw, SU, and DGC spermatozoa was limited to a restricted small area located in the middle nuclear region, toward its apical side (Fig. 6a, b). Analysis also revealed several changes in the chromosome order within the sperm nucleus.

The results based on the basal–apical criterion consistently demonstrated that, across all the analyzed fractions, centromeres of chromosomes X and Y were predominantly located in the apical region, while centromeres of chromosomes 4 and 8 were positioned at the basal nuclear region. Notably, the apical localization of centromere of chromosome X was observed in all analyzed samples (Fig. 6c).

Generally, according to the “centre–periphery” criterion, chromosome 4 consistently occupied the most peripheral position across all the analyzed sperm fractions, while chromosome 18 preferred positioning at mid-depth level. In contrast, chromosomes X and Y in raw and DGC spermatozoa were located most centrally, deep within the sperm nucleus, and chromosomes 7 and 9 in SU spermatozoa (Fig. 6a and Table 5).

It was also observed that chromosomes 4 and 8 and chromosomes 7 and 9 colocalized and assumed a position in the middle nuclear region in raw, SU, and DGC spermatozoa (Fig. 6a, c) and also in each individual case (Additional file 7).

We have observed that all analyzed chromosomes exhibited various changes in their localization within SU or DGC spermatozoa, except for chromosome 7, which retained its stable position across all the sperm fractions (Table 6). Detailed descriptions of these changes are provided below.

### Swim up fraction (SU)

In SU spermatozoa, chromosome 4 was repositioned from central area toward the apical nuclear region when compared with raw spermatozoa (*p* = 0.0079) (Fig. 6a). Centromeres of both chromosomes X and Y were repositioned from central nuclear area toward the peripheral when compared with raw spermatozoa (*p* = 0.0047 and *p* = 0.0011, respectively) and DGC spermatozoa (*p* = 0.0021 and *p* = 0.0071, respectively) (Fig. 6a).

Furthermore, centromere of chromosome 18 from unselected and X-spermatozoa shifted from central toward the peripheral area in SU spermatozoa, when compared with raw spermatozoa (*p* < 0.0001) and DGC spermatozoa (*p* = 0.0467 and *p* = 0.036, respectively) (Fig. 6a), whereas in Y-spermatozoa, the shift of centromere of chromosome 18 was not statistically significant (*p* > 0.05) (Fig. 6a). There were no statistically significant changes in positioning of centromeres of chromosomes 7, 8, and 9 (*p* > 0.05) (Fig. 6a).

### Percoll density gradient centrifugation fraction (DGC)

Similarly to the results obtained for the SU fraction, also for DGC spermatozoa, chromosome 4 was repositioned from central toward the apical nuclear region when compared with raw spermatozoa (*p* < 0.0001) (Fig. 6a). Chromosome 8 shifted from central area toward the nuclear apical and peripheral area (*p* < 0.0001), while chromosome 9 shifted from central toward the peripheral nuclear region when compared with raw spermatozoa (*p* = 0.0118) (Fig. 6a).

### Radial positioning in differentially methylated (5mC) and hydroxymethylated (5hmC) spermatozoa

After evaluating the positioning in differentially methylated (5mC) and hydroxymethylated (5hmC) spermatozoa, there were no significant differences in the positioning of all analyzed chromosomes within the sperm fractions (*p* > 0.05) (Table 5).

### Chromocenters

Hierarchical Ward clustering showed that, in raw spermatozoa, chromosomes were localized in three chromocenters: pairwise (4 and 8), in a group (7, 9, 18, and Y), or remaining single (X) (Fig. 6c, e). In comparison, in SU spermatozoa, chromosomes were localized in four chromocenters: pairwise (4 and 8, 7 and 9, and X and Y) or remained single [18], while in DGC spermatozoa, chromosomes were localized in two groups (4, 8, and 18 and 7, 9, Y, and X) (Fig. 6e). In SU and DGC spermatozoa, the chromocenter occupied a more restricted area than in raw sperm (Fig. 6b–e). Our findings suggest the presence of distinct chromocenter fragments in each sperm fraction and highlight the differences in chromosomal distribution.

### Distances between the chromosomes’ centromeres

**Fig. 7:**
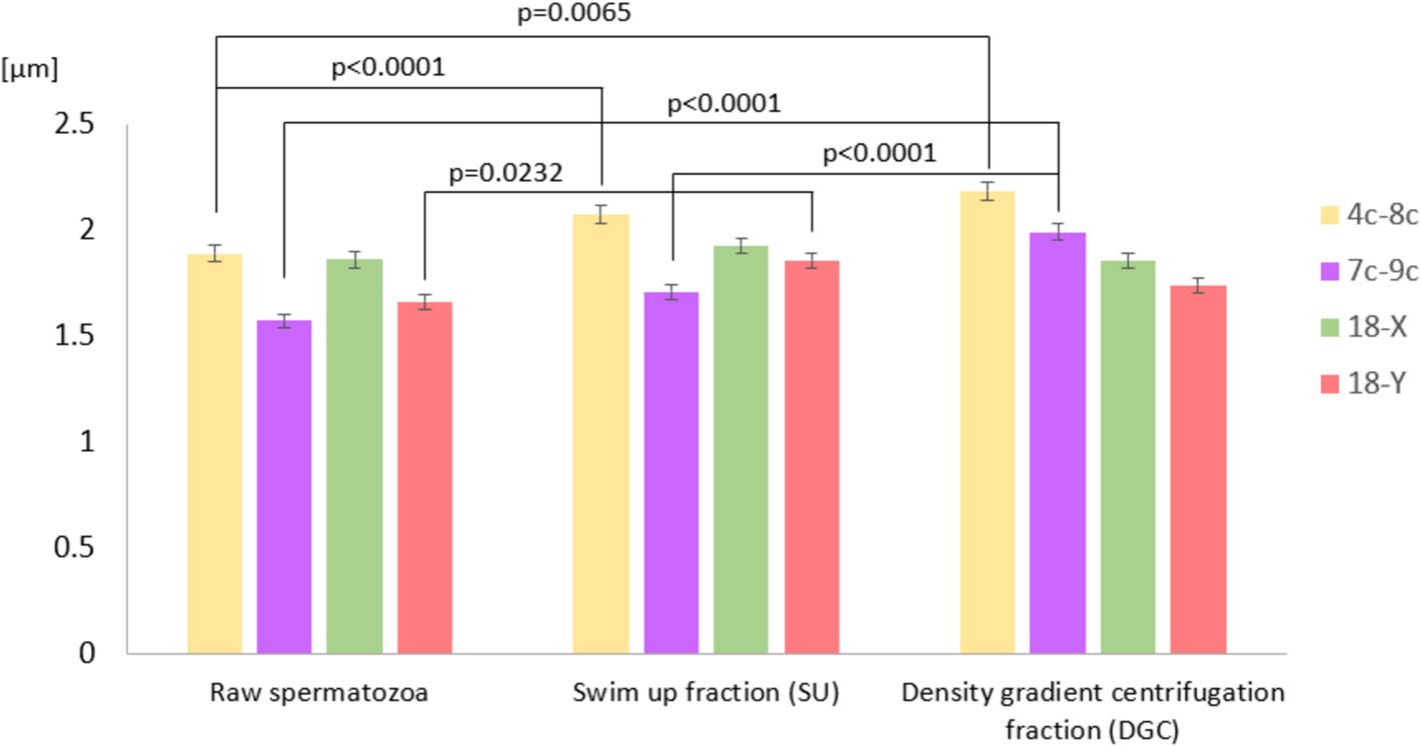
Distances between the centromeres of chromosomes 4 versus 8, 7 versus 9, 18 versus X, and 18 versus Y in raw spermatozoa, swim up fraction (SU), and density gradient centrifugation fraction (DGC) in *n* = 5 normozoospermic cases (K1–K5). Statistical significance was considered at *p* < 0.05

The average distances between centromeres of selected chromosome pairs (4 versus 8, 7 versus 9, 18 versus X, 18 versus Y) were analyzed across the examined sperm fractions. Pairs 4/8 and 7/9 were chosen on the basis of our previous observations of frequent colocalization [23, 26, 118], whereas chromosomes 18, X, and Y were most commonly studied in sperm nuclear organization [23, 26, 28, 106, 109–116, 118–125]. For the first time, we additionally distinguished the X-bearing spermatozoa from Y-bearing spermatozoa for 18 chromosome’s topology evaluation to get an answer regarding possible nuclear organization differences. The results of this evaluation are presented in Table 7, Fig. 7, and Additional file 8. In line with previous reports of possible interindividual variability in localization of chromosomes [23, 26, 111, 112], also in this study we examined this aspect (Additional file 6).

**Table 7:** Distances between the centromeres of chromosomes 4 and 8, 7 and 9, 18 and X, and 18 and Y in raw spermatozoa and good-quality fractions (SU and DGC)

The average distance between chromosomes [µm]				
Chromosomes		Raw spermatozoa	SU fraction	DGC fraction
4–8	Mean	1.888	2.073**	2.184**
	SE	0.03797	0.03953	0.04091
7–9	Mean	1.571	1.707	1.989**#
	SE	0.034	0.03674	0.03991
18-X	Mean	1.859	1.926	1.853
	SE	0.04028	0.03576	0.03439
18-Y	Mean	1.662	1.858*	1.739
	SE	0.03512	0.03596	0.03566

^*^ Values different significantly from the mean value of raw spermatozoa (*p* < 0.05)

^**^ Values different significantly from the mean value of raw spermatozoa (*p* < 0.01)

^#^ Value different significantly from mean value of swim up fraction (*p* < 0.01)

In both SU and DGC spermatozoa, the distance between the centromeres of chromosomes 4 and 8 increased when compared with raw spermatozoa, by 1.1-fold in SU spermatozoa (*p* = 0.0065) and by 1.16-fold in DGC spermatozoa (*p* < 0.0001) (Fig. 7). Chromosomes 4 and 8 consistently colocalized in all three analyzed sperm populations, and the interindividual variability remained at the negligible level (with 0 out of 5 variable cases and 2 out of 5 noted in SU or DGC spermatozoa) (Additional file 6).

When considering the distance between centromeres of chromosomes 7 and 9, it was found that, in DGC spermatozoa, this value was 1.27-fold higher than in raw spermatozoa (*p* < 0.0001), and 1.17-fold higher when compared with SU spermatozoa (*p* < 0.0001) (Fig. 7). Chromosomes 7 and 9 were located similarly: each showed variability in 1/5 cases in raw spermatozoa, 3/5 or 4/5 cases in SU spermatozoa, respectively, and 2/5 in DGC spermatozoa. Despite high variability in SU spermatozoa, the colocalization of chromosomes 7 and 9 remained stable (Additional file 6; Fig. 6a).

In case of X-spermatozoa, the variability of chromosome 18 position increased from none in raw spermatozoa, to 1/5 cases in SU spermatozoa, and 2/5 in DGC spermatozoa, while the variability of chromosome X positioning remained at an intermediate level (3/5 cases in raw spermatozoa and SU spermatozoa and 2/5 in DGC spermatozoa) (Additional file 6). This observation may suggest some kind of linkage between reciprocal positioning of chromosomes 18 and X, which can be supported by the fact of the unchanged distances between them (*p* > 0.05) (Fig. 7).

A distance between centromeres of chromosomes 18 and Y was 1.12-fold higher in SU spermatozoa than in raw spermatozoa (*p* = 0.0232), while in DGC spermatozoa the distances remained unchanged (*p* > 0.05) (Fig. 7). For the Y chromosome, we documented that both fractioning methods had a positive influence in terms of stabilizing the Y chromosome position (from 3/5 cases in raw spermatozoa, 2/5 in SU spermatozoa, to 1/5 in DGC spermatozoa) (Additional file 6). In Y-spermatozoa, chromosome 18 displayed almost no variability, what seems to underline the independent positioning pattern of those chromosomes, as also confirmed by the changed distances between them, depending on the observed sperm fraction (1.12-fold higher in SU spermatozoa than in raw spermatozoa (*p* = 0.0232) (Fig. 7; Additional file 6).

There were no significant differences in distances between spermatozoa with high or low 5mC and 5hmC levels (*p* > 0.05), independently of the analyzed sperm fraction.

## Discussion

This is the first study focusing on differences in chromosomal positioning within the sperm nucleus in high-quality spermatozoa selected via swim-up (SU) or centrifugation in a density gradient (DGC), techniques still used in some ART centers. A sequential staining algorithm was applied to analyze both chromosomal localization, as well as the level of epigenetic modifications, specifically DNA methylation (5mC) and hydroxymethylation (5hmC), on the same individual sperm. The experimental algorithm was conducted step by step and involved: (i) the separation of high-quality fractions, (ii) topological measurements, and (iii) the assessment of global levels of sperm DNA 5mC and 5hmC, with already determined positioning of evaluated chromosomes. This integrated approach allowed us to correlate the spatial organization of chromosomes with the epigenetic profile of spermatozoa across different sperm quality fractions. By combining all molecular data obtained, including also characteristics of sperm chromatin integrity (chromatin protamination and DNA fragmentation), the study also focused on sperm quality indicators, in the paternal chromosomal context.

Initially, we performed a qualitative assessment of sperm chromatin to determine the structural integrity and overall quality of the sperm DNA in evaluated males. Our findings revealed that the frequency of high-quality spermatozoa with properly protaminated chromatin was significantly higher in selected good-quality sperm fractions (SU and DGC) compared with nonfractionated, native ejaculated (raw) samples. This finding aligns with previous studies demonstrating reduced histone retention in gradient-selected sperm fractions [69]. It was also shown that increased chromatin condensation may protect critical regions of the sperm genome, including genes involved in early embryonal development. Regions retaining histones may also harbor genes necessary for these early development processes [126, 127]. It has been documented that imbalance in the ratio of protamines to the remaining histones has been implicated in male infertility [22–24], revealing a reduction of sperm quality or induction of sperm DNA damage [128–131], and ultimately leading to a breakdown of the fertilization process or a decreased fertilization rate [132] and/or embryonic development [68, 86, 133]. It was also shown that poor sperm chromatin protamination negatively affects fertilization ICSI outcome rates involving gametes from healthy donors [126]. These data followed by findings in our study emphasize the importance of chromatin integrity and proper DNA protamination for successful fertilization and embryogenesis and highlight the need for careful sperm selection.

In our study, no significant changes were observed in SDF levels across the analyzed intact and fractionated sperm samples using the TUNEL assay, which detects both single-stranded (ssDNA) and double-stranded DNA (dsDNA) breaks. This result may be attributed to the fact that the study population exclusively consisted of control men without a history of reproductive failures, thus the demonstrated low baseline levels of DNA damage seem to be reasonable. On the other hand, in previous studies performed with infertile patient groups, altered levels of ssDNA and dsDNA breaks were clearly documented [69, 134]. Interestingly and as still widely discussed, there are ongoing discrepancies concerning methods used for sperm DNA fragmentation, supported by data originated from different laboratories. However, it has been shown that, in motile spermatozoa populations selected by DGC, from infertile patients with normal and abnormal semen parameters, there was a significant decrease in the frequency of sperm with damaged DNA [135–137], whereas in the applied SU method the recovered sperm showed no significant improvement [138]. In contrast, other studies found that the frequency of sperm with fragmented DNA was reduced significantly after SU treatment but not after DGC, compared with raw spermatozoa of infertile normozoospermic or oligozoospermic patients [100, 139]. In one study, DNA fragmentation was lower after DGC and SU procedures [137].

In our study, we also applied the Acridine Orange (AO) test, which specifically detects single-stranded DNA breaks. We have documented statistically significant differences between SU and DGC recovered spermatozoa, which exhibited decreased levels of ssDNA fragmentation when compared with raw spermatozoa. These observations may indicate that the efficacy of SDF reduction may vary, being dependent on the type of assay used (relationship to a particular type of DNA damage) and studied male population, highlighting the need for standardization of approaches used for this purpose. During ART procedures, especially in ICSI, insemination or microinjection makes use of spermatozoa with apparently normal morphology and 4D motility, being nevertheless a random process that may result in the unintended use of DNA-damaged spermatozoa, as molecular changes are not visible microscopically [103]. Therefore, it is important to assess the degree of human SDF, as this parameter has been linked to fertilization outcomes, embryo development, pregnancy success, and reduction of the risk of transmitting genetic damage to offspring [69, 103, 103, 119, 131, 134–136, 138–141]. What is also crucial is the fact that spermatozoa lack DNA repair mechanisms and if the level of sperm DNA damage exceeds the oocyte’s capacity to repair it, then embryos might undergo apoptosis [26, 142].

The analysis of global levels of DNA methylation (5mC) and hydroxymethylation (5hmC) revealed high levels of both epimarks in SU and DGC spermatozoa compared with raw spermatozoa. A statistically significant difference was observed exclusively in SU spermatozoa, in which the levels were approximately 12% higher than in raw spermatozoa. In DGC spermatozoa, 5mC and 5hmC levels were still approximately 6.5% higher than those in raw spermatozoa, indicating a positive potential trend, albeit without statistical significance. In previous studies, it was shown that spermatozoa from fractionated samples may reflect different methylation patterns [69, 143, 144]. Similarly, in fertile normozoospermic males, 5mC values were higher than in infertile patients [107, 145]. Changes in gene methylation patterns were also documented in normozoospermic and infertile males [146, 147]. Previous reports indicated that pregnancy rates were higher when sperm DNA methylation was above a certain threshold [148, 149]. Thus, it can be suggested that improved sperm selection techniques may be able to select a sperm population with a proper epigenetic profile. Future studies need to determine what is considered normal in epigenetics and establish the real risks that are important for embryo development and the health of offspring [143]. As our study focused on global DNA methylation and hydroxymethylation in a group of normozoospermic men without known fertility problems, the lack of significant differences may be due to the fact that this group was quite homogeneous. Wider differences in methylation patterns might be found in more diverse populations, especially those including men with impaired spermatogenesis or reproductive failures [107, 108].

The nuclear sperm architecture has been supposed to influence early embryonic development [150–153], including the later zygote stages [123, 153]. It was suggested that abnormal chromosomal positioning within the sperm nucleus can have a negative impact on fertilization and early embryogenesis [154], highlighting the importance of chromatin organization in reproductive success. Changes in sperm sex chromosome positioning have been reported in men with impaired semen parameters [125], while abnormal nuclear organization has been associated with defective sperm head morphology [155]. Disturbances in the nuclear positioning of chromosomes may be linked to compromised spermatogenesis, suggesting that the structural integrity and position of chromosomal territories are crucial for normal sperm function [119]. In spermatozoa, centromeres of chromosomes have a tendency to aggregate when composing chromocenters [121, 152, 156, 157]. It was shown that, in immotile spermatozoa, that chromocenter formation was disrupted [121]. In previous studies on normozoospermic males, it was demonstrated that less than 10% of spermatozoa contained a single chromocenter, with a common tendency to be located in the central region [121, 125, 154]. There are also data revealing the existence of 1–3 chromocenters in 73% of spermatozoa from normozoospermic males [121]. In comparison, in our study, which focused on normozoospermic males, the number of chromocenters varied between 2 and 4. This difference can be suggested to be a result of various number of chromosomes evaluated—Alladin et al. performed FISH with pan-centromeric probes for all chromosomes (but not specifically labeled), while we evaluated only seven of them [121]. In our study, a reduction of the overall chromocenter area was also observed, which is not surprising as our data were focused on selected, good-quality sperm fractions, the most representative ones for the fertilization process, and therefore treated as the reference ones. It has to be underlined that our study is the first observation of this kind, revealing the specific topology of chromosomes in good-quality spermatozoa. Importantly, it was observed previously that, in males with disrupted spermatogenesis, a wider area of chromocenters was documented [109, 111, 112, 121]. Ioannou et al. also suggested that specific chromosomes may preferentially contribute to the formation of individual chromocenters, and more fragmented arrangement of chromocenters may play a key role in the ordered release of paternal chromosomes after fertilization [8].

Our findings also confirm previously published data that centromeres of chromosomes have a defined and stable position in raw spermatozoa [12, 23, 112]. In our study, chromosome 4 relocated from central toward the apical sperm region in both SU and DGC spermatozoa in comparison with raw spermatozoa, while chromosomes 18, X, and Y shifted from central area to the nuclear periphery. Additionally, in selected DGC spermatozoa, chromosome 8 was repositioned from central area toward the apical and peripheral nuclear region, with the chromosomes 9 and Y localization changed from the central area in the direction of the periphery. These findings align with previous research suggesting that chromosomal size and gene density influence their own spatial organization within the sperm nucleus. Chromosome 4, being one of the larger human chromosomes, may be more susceptible to mechanical forces during topological reorganization [118, 122, 158]. It was also observed here that chromosomes 4 and 8, despite their individual repositioning patterns, colocalized and assumed a position in the middle nuclear region in raw, SU, or DGC spermatozoa, as in previously published topological studies [23, 118]. Chromosomes 7 and 9 have been shown to localize primarily in the middle or peripheral sperm nuclear regions [12, 118], and their stable positioning in the middle nuclear region, as also observed in the present study, may suggest their role in maintaining nuclear architecture. Further studies are needed to confirm this feature in other medium-sized and gene-rich chromosomes.

We further observed that chromosomes 18, X, and Y aligned in a consistent order across all the analyzed sperm fractions, from the basal to the apical nuclear regions, following the sequence 18, Y, and X, as detected in previous studies [23, 122]. This chromosome positioning order might reflect functional significance, potentially linked to chromosomal properties such as size, gene density, or their involvement in early embryonic development [35, 152].

Additionally, our observation that chromosome 18 is preferentially positioned at the nuclear periphery is consistent with earlier studies indicating a preferential peripheral localization for smaller, gene-poor chromosomes [12, 118, 122, 150, 152]. The observed pattern supports the theory that chromosomes with fewer genes may be positioned in regions of the nucleus that are less transcriptionally active after fertilization, reflecting their low contribution to early embryonic gene expression [152, 159]. Furthermore, in SU X-spermatozoa, chromosomes 18 and X shifted their position from the central area toward the periphery of sperm nucleus compared with raw sperm fraction, resulting in no change in the distance between these chromosomes. In contrast, in SU Y-spermatozoa, chromosome 18 remained in unchanged position, while chromosome Y shifted also from central area toward periphery, leading to an increase in the distance between these chromosomes, compared with raw sperm fraction. Interestingly, in Y-chromosome spermatozoa (raw, SU, and DGC), chromosome 18 showed minimal positional variability, which seems to highlight the independent positioning pattern of chromosomes 18 and Y. In contrast, in X-spermatozoa (SU, DGC), positioning variability of chromosome 18 increased regarding raw spermatozoa, while variability of chromosome X positioning remained moderately variable (in 2–3 out of 5 cases). The rising variability of chromosome 18 in X-spermatozoa (SU, DGC), alongside consistent X chromosome positioning and unchanged distances between 18 and X, may point to some kind of linkage between reciprocal positioning between these chromosomes within the sperm nucleus.

Our findings also show that sex chromosomes in high-quality spermatozoa tend to take a position in the apical nuclear region, which is known to be involved in the initial ooplasm interaction during fertilization, followed by chromatin reorganization of the paternal genome and early transcriptional activity at the early stages of embryonal development. Therefore, a specific order of the chromosomes carrying particular genes critical for those initial fertilization events seems to add some kind of epigenetic regulative layer at the chromosomal level [12, 26, 27, 109, 111, 112, 118, 120, 121, 123–125, 151, 156, 160–164]. Importantly, previously reported alterations in the positioning of sex chromosomes have been documented in cases with severe types of infertility, in samples with reduced semen quality, such as low sperm count or motility [119]. In our study, the centromere of chromosome Y also shifted toward the peripheral region from central area in SU spermatozoa, in contrast to raw spermatozoa. In previous studies, the Y-chromosome was shown to exhibit variability in centromere positioning, which may reflect the smaller size, lower gene density, and specific gene functions of the Y-chromosome [23, 111, 112, 159]. The Y-chromosome is known to exhibit the most interindividual differences in localization within the sperm nucleus [23, 26, 111, 112]. In our study, it was shown that both fractioning methods effectively reduced the variability of Y-chromosome positioning.

In our study, an interesting issue was observed for case K2, in which spermatozoa fractionation improved sperm chromatin protamination status the most, among all evaluated cases (raw spermatozoa 65.42% versus SU fraction 95.17% versus DGC fraction 96.16%; Additional file 3). Simultaneously, only in K2, a chromosome positioning shift was observed between raw spermatozoa and both good-quality sperm fractions, for each of the evaluated chromosomes (Additional file 7). These observations seem to clearly point to the reasonable application of sperm fractioning methods, necessary for selection of spermatozoa with the highest quality, and their role in enhancing both chromatin quality and chromosomal topology.

The choice of sperm fractioning methods for this study was also driven by their relevance to ART treatments, particularly IVF and ICSI, where spermatozoa are used after initial semen preparation (selection) to improve fertilization outcomes. However, ICSI has raised concerns because it bypasses natural selection mechanisms, potentially increasing the risk of microinjecting a sperm with chromosomal aberrations or fragmented DNA, which are not detectable under standard microscopic evaluation by the embryologist [103, 165]. One of the major differences between natural fertilization versus ICSI is the absence of sperm modifications (e.g., acrosomal reaction, membrane fusion), which occur at fertilization, which can lead to delayed decondensation of the sperm nucleus owing to the presence of an intact cytoplasmic membrane, which delays ooplasm contact with the nuclear envelope [164, 166]. This is associated with increased fertilization abnormalities and failures following ICSI, especially if the oocyte is not artificially activated to overcome sperm–oocyte fusion [12, 164, 167]. Moreover, human sperm microinjected into hamster oocytes showed that aberrant nuclear decondensation started from the basal nuclear region [164]. If sperm nuclei undergo delayed or hindered decondensation during male pronucleus development, the apical localization of the sex chromosomes may contribute to an increased risk of sex chromosome aberrations in ICSI-conceived offspring. This could occur owing to delayed entry into the S-phase, which may result in mitotic errors during the first cleavage [28, 164]. This hypothesis can be supported by the fact that an increased rate of sex chromosomal aberrations has been reported in progeny conceived by ICSI [168, 169]. It is interesting that, in our study, the X and Y chromosomes in the good-quality sperm fractions remained in the apical sperm region, but were repositioned to the nuclear periphery from central area, which seems to elevate and strengthen the importance of the chromosomal organization context. This hypothesis requires further investigation, also including other methods of sperm selection, as well as high-resolution mapping of the sperm genome and epigenome on the basis of chromatin capture.

## Conclusions

This study demonstrates that selection of high-quality sperm, on the basis of motility and morphology, significantly enhanced chromatin protamination, reduced sperm ssDNA fragmentation, increased 5mC and 5hmC levels, and showed distinct patterns of chromosome positioning. Chromosome repositioning was documented, particularly for sex chromosomes, which shifted toward the sperm nuclear apical-periphery, which is crucial for the initial interaction with the ooplasm during fertilization. The findings highlight the importance of sperm fractionation methods in the chromosomal and chromatin context concerning selection of spermatozoa with good characteristics for assisted reproductive treatments. This targeted selection highlights the importance of further research on chromatin interactions and dynamics in spermatozoa, and their implications for fertilization and embryo development, and also to refine ART protocols for improved embryological and clinical outcomes.

## Supplementary Information

Additional file 1

Additional file 2

Additional file 3

Additional file 4

Additional file 5

Additional file 6

Additional file 7

Additional file 8

Additional file 9

## Data Availability

All the data generated during this study are included in this manuscript, and the raw data basis is deposited in a Zenodo repository (10.5281/zenodo.15599300).

## Abbreviations

5hmC: DNA hydroxymethylation
5mC: DNA methylation
AB: Aniline Blue
AO: Acridine Orange
ART: Assisted reproductive technology
BGR: Blue, green, and red filter
BSA: Bovine serum albumin
ChIA-Drop: Chromatin interaction analysis with droplet-based high-throughput sequencing
CT: Chromosome territory
DAPI: 4′,6-Diamidino-2-phenylindole
DGC: Density gradient centrifugation
dsDNA: Double-stranded DNA
DTT: Dithiothreitol
FISH: Fluorescent in situ hybridization
GAM: Genome architecture mapping
Hi-C: High-throughput chromosome conformation capture
ICSI: Intracytoplasmic sperm injection
IF: Immunofluorescence staining
IMSI: Intracytoplasmic morphologically selected sperm injection
IVF: In vitro fertilization
PBS: Phosphate-buffered saline
PBST: PBS + Triton X-100
SDF: Sperm DNA fragmentation
SpG: Spectrum Green
SpO: Spectrum Orange
SPRITE: Split-pool recognition of interactions by tag extension
SSC: Saline sodium citrate
ssDNA: Single-stranded DNA
SU: Swim up fraction
TAD: Topologically associating domain
Tet: Ten–eleven translocation proteins
TLC: Thin-layer chromatography
TP: Transition protein
WHO: World Health Organization
XCI: X chromosome inactivation
