# Supplementary material for: Impact of sperm fractionation on chromosome positioning, chromatin integrity, DNA methylation, and hydroxymethylation level
Source: Cell Mol Biol Lett. 2025 Dec 23;30:146. doi: 10.1186/s11658-025-00830-7 (PMC12743405; doi:10.1186/s11658-025-00830-7)
Supplement: Supplementary file 1 — Additional file 1 [file 11658_2025_830_MOESM1_ESM.pdf]

Chromosome	Catalogue Number	Locus	Chromosome region	DNA class	Spectrum
4	LPE 004G	D4Z1	4p11.1-q11.1	α-satellite	Green
7	LPE 007G	D7Z1	7p11.1-q11.1	α-satellite	Green
8	LPE 008R	D8Z2	8p11.1-q11.1	α-satellite	Orange
9	LPE 009R	D9Z3	9q12	satellite III	Orange
18	LPA 004	D18Z1	18p11.1-q11.1	α-satellite	Aqua
X	LPE 0XG	DXZ1	Xp11.1-q11.1	α-satellite	Green
Y	LPE 0YcR	DYZ3	Yp11.1-q11.1	α-satellite	Orange
