# Supplementary material for: Impact of sperm fractionation on chromosome positioning, chromatin integrity, DNA methylation, and hydroxymethylation level
Source: Cell Mol Biol Lett. 2025 Dec 23;30:146. doi: 10.1186/s11658-025-00830-7 (PMC12743405; doi:10.1186/s11658-025-00830-7)
Supplement: Supplementary file 2 — Additional file 2 [file 11658_2025_830_MOESM2_ESM.pdf]

Chromosome	Size (Mb)	Protein-coding genes	Non-coding genes	Pseudogenes	Gene density (protein coding genes/Mb)
4	190.2	759	2030	755	4.0
7	159.3	1016	1999	904	6.4
8	145.1	704	1895	638	4.9
9	138.4	777	1705	689	5.6
18	80.4	267	1073	264	3.3
X	156.0	866	1188	886	5.6
Y	57.2	61	219	392	1.1
