# Supplementary figures and images for: Impact of sperm fractionation on chromosome positioning, chromatin integrity, DNA methylation, and hydroxymethylation level
Source: Cell Mol Biol Lett. 2025 Dec 23;30:146. doi: 10.1186/s11658-025-00830-7 (PMC12743405; doi:10.1186/s11658-025-00830-7)

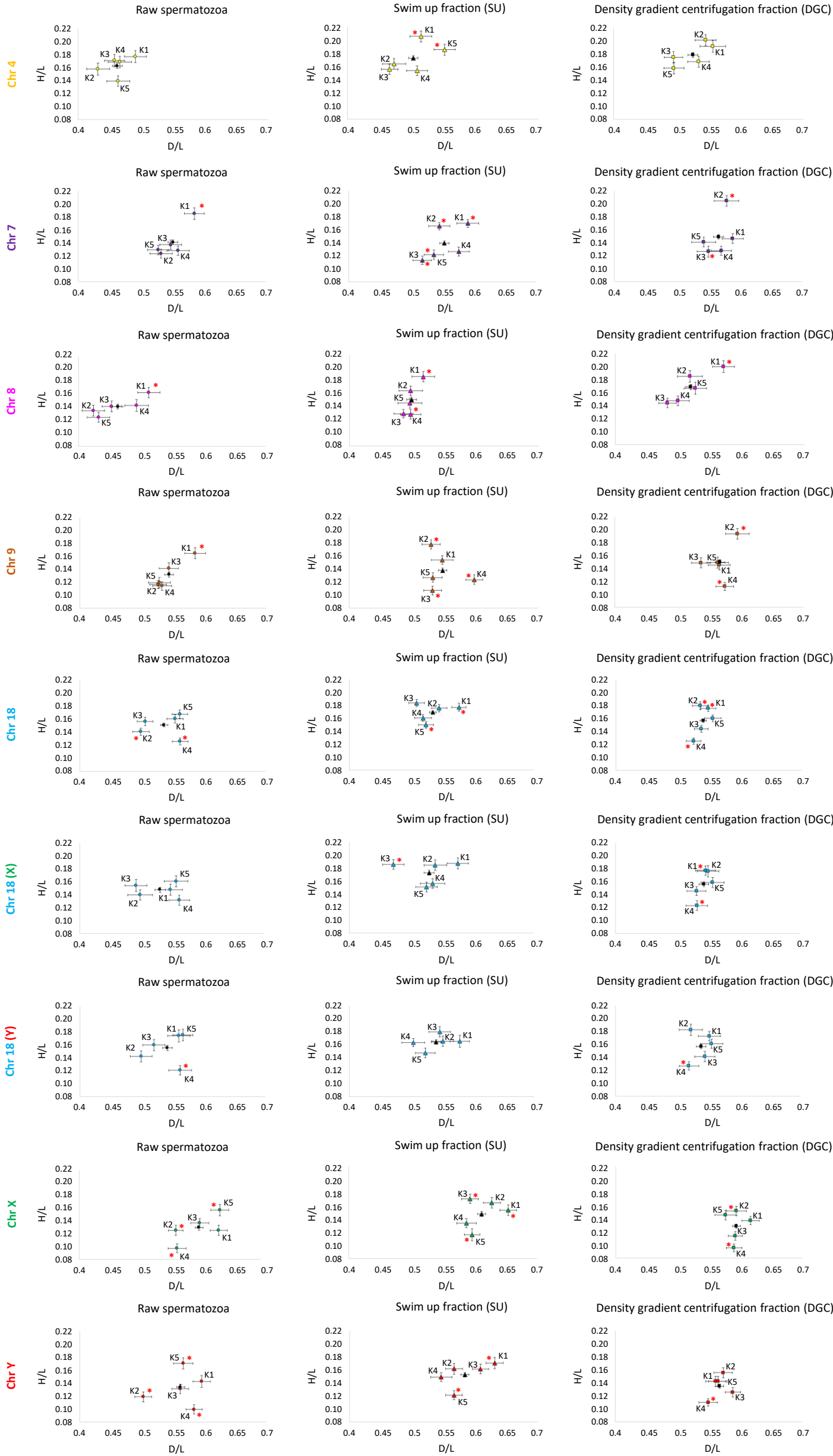

Supplement: Supplementary file 6 — Additional file 6 [file 11658_2025_830_MOESM6_ESM.pdf]

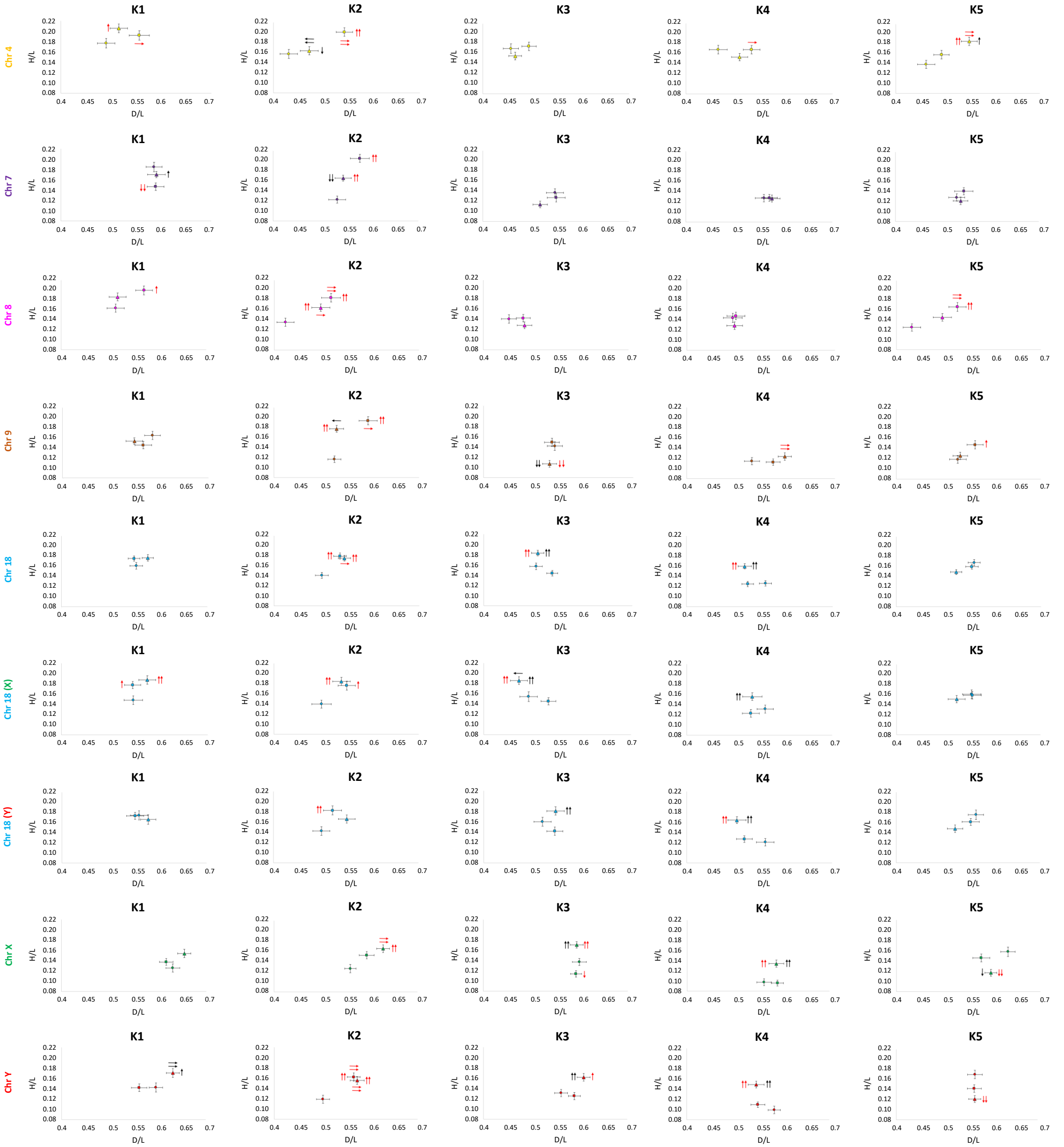

Supplement: Supplementary file 7 — Additional file 7 [file 11658_2025_830_MOESM7_ESM.pdf]
