# Supplementary material for: Impact of sperm fractionation on chromosome positioning, chromatin integrity, DNA methylation, and hydroxymethylation level
Source: Cell Mol Biol Lett. 2025 Dec 23;30:146. doi: 10.1186/s11658-025-00830-7 (PMC12743405; doi:10.1186/s11658-025-00830-7)
Supplement: Supplementary file 9 — Additional file 9 [file 11658_2025_830_MOESM9_ESM.docx]

Additional file 1 .pdf FISH probes used for experiments (Cytocell, Cambridge, UK).

Additional file 2 .pdf Size, number of genes and gene density of chromosomes analysed (according to Ensembl database <https://www.ensembl.org/Homo_sapiens/Location/Genome>)

Additional file 3 .xlsx Individual data for results of sperm chromatin integrity evaluation in non-fractionated sperm population (raw spermatozoa) and good-quality fractions (swim up fraction (SU) and Percoll density gradient centrifugation fraction (DGC)) in each evaluated case.

Additional file 4 .xlsx Individual data for linear positioning of centromeres of chromosomes: 4, 7, 8, 9, 18, X and Y in spermatozoa in each evaluated case.

Additional file 5 .xlsx Individual data for radial positioning of centromeres of chromosomes: 4, 7, 8, 9, 18, X and Y in spermatozoa in each evaluated case.

Additional file 6 .pdf Radial positioning of the each of examined centromeres (4, 7, 8, 9, 18, X, Y) separately within the sperm nucleus in raw spermatozoa, swim up fraction (SU) and density gradient centrifugation fraction (DGC), according to data in Additional file 3 (circle: raw spermatozoa, triangle: swim up fraction (SU), square: density gradient centrifugation fraction (DGC)). Localizations that differ significantly from the raw spermatozoa mean value were indicated by red arrows: double arrow for p ≤ 0.01, single arrow for p ≤ 0.05. Statistically significant differences between SU and DGC fractions were indicated by black arrows: double arrow for p ≤ 0.01, single arrow for 0.01 < p ≤ 0.05. Arrows also indicate the direction of the observed shift (repositioning) of centromeres. Bars show standard errors (SE).

Additional file 7 .pdf Individual data for radial positioning of the examined centromeres (4, 7, 8, 9, 18, X, Y) within the sperm nucleus in raw spermatozoa, swim up fraction (SU) and density gradient centrifugation fraction (DGC) in each evaluated case, according to the data in Additional file 3 (circle: raw spermatozoa, triangle: swim up fraction (SU), square: density gradient centrifugation fraction (DGC)). The mean control values are marked in black. Red asterisks indicate statistically significant differences compared to the mean values. Bars show standard errors (SE).

Additional file 8 .xlsx Distances between the centromeres of chromosomal pairs: 4 and 8, 7 and 9, 18 and X, 18 and Y in raw spermatozoa and good-quality fractions (swim up fraction (SU) and density gradient centrifugation fraction (DGC)) in each evaluated case.
